# Evolutionary profiling reveals the heterogeneous origins of classes of human disease genes: implications for modeling disease genetics in animals

**DOI:** 10.1186/s12862-014-0212-1

**Published:** 2014-10-04

**Authors:** Evan K Maxwell, Christine E Schnitzler, Paul Havlak, Nicholas H Putnam, Anh-Dao Nguyen, R Travis Moreland, Andreas D Baxevanis

**Affiliations:** Computational and Statistical Genomics Branch, Division of Intramural Research, National Human Genome Research, National Institutes of Health, Bethesda, MD 20892 USA; Bioinformatics Program, Boston University, Boston, MA 02215 USA; Department of Ecology and Evolutionary Biology, Rice University, Houston, Texas 77005 USA; Biomedical Informatics Core, College of Medicine, Texas A&M Health Science Center, Houston, Texas 77030 USA

**Keywords:** Model organism selection, Human disease genes, Evolutionary genetics, Comparative genomics

## Abstract

**Background:**

The recent expansion of whole-genome sequence data available from diverse animal lineages provides an opportunity to investigate the evolutionary origins of specific classes of human disease genes. Previous studies have observed that human disease genes are of particularly ancient origin. While this suggests that many animal species have the potential to serve as feasible models for research on genes responsible for human disease, it is unclear whether this pattern has meaningful implications and whether it prevails for every class of human disease.

**Results:**

We used a comparative genomics approach encompassing a broad phylogenetic range of animals with sequenced genomes to determine the evolutionary patterns exhibited by human genes associated with different classes of disease. Our results support previous claims that most human disease genes are of ancient origin but, more importantly, we also demonstrate that several specific disease classes have a significantly large proportion of genes that emerged relatively recently within the metazoans and/or vertebrates. An independent assessment of the synonymous to non-synonymous substitution rates of human disease genes found in mammals reveals that disease classes that arose more recently also display unexpected rates of purifying selection between their mammalian and human counterparts.

**Conclusions:**

Our results reveal the heterogeneity underlying the evolutionary origins of (and selective pressures on) different classes of human disease genes. For example, some disease gene classes appear to be of uncommonly recent (*i.e.,* vertebrate-specific) origin and, as a whole, have been evolving at a faster rate within mammals than the majority of disease classes having more ancient origins. The novel patterns that we have identified may provide new insight into cases where studies using traditional animal models were unable to produce results that translated to humans. Conversely, we note that the larger set of disease classes do have ancient origins, suggesting that many non-traditional animal models have the potential to be useful for studying many human disease genes. Taken together, these findings emphasize why model organism selection should be done on a disease-by-disease basis, with evolutionary profiles in mind.

**Electronic supplementary material:**

The online version of this article (doi:10.1186/s12862-014-0212-1) contains supplementary material, which is available to authorized users.

## Background

The set of human genes implicated in Mendelian diseases are of particular interest in biomedical research. These “disease genes” contain mutations that increase susceptibility to a disease phenotype, but are tolerated well enough as to not cause lethality in early developmental stages. Studies have demonstrated that disease genes are a non-random subset of all human genes [1-6]. For example, human disease genes tend to be non-essential, having relatively few interacting partners; as a result, disease genes are often located on the periphery of gene networks [1,2]. From an evolutionary perspective, human disease genes tend to have particularly ancient origins [3-6], suggesting that disease-causing mutations are more often identified in “older” genes. Human disease genes also display unique patterns of purifying selection, duplication history, and tissue-specific expression [1,4,6].

The implications of these observations in the context of how human disease research is conducted are not well understood. One proposition is that the tendency for disease genes to be of ancient origins implies that they are often functionally conserved across many animal lineages. Consequently, it may be possible to study disease genes in a broad spectrum of animal models. For example, a previous study estimated that over 90% of disease genes emerged prior to the divergence of bilaterally symmetrical (bilaterian) animals [3]. This evolutionary divergence, which dates back over 600 million years [7], is marked by rapid innovation that gave rise to the vast majority of animal species living today. Another study noted that ~44% of a curated subset of disease genes were found to have orthologs in the yeast *Saccharomyces cerevisiae* [8,9].

The use of traditional model organisms that are relatively closely related to humans (including primates, mice, and, more recently, zebrafish) has been quite successful in yielding results that can translate to humans [10-15], but more distantly related animals have also been utilized for studying various human disease genes and diseases. Pharmaceutical companies have successfully used *Caenorhabditis elegans* [16] and *Drosophila melanogaster* [17] in drug discovery research. The sea anemone *Nematostella vectensis* is becoming recognized as a strikingly useful model organism, despite being a non-bilaterian animal even more remotely related to humans than worms and flies [9,18,19]. Most recently, major expansions to the inventory of whole-genome sequences from species across the animal tree have fueled the effort to identify and develop new model systems, with some of these species beginning to demonstrate real potential for the study of human disease [18,20-22].

In part, efforts to introduce new model systems to the standard experimental repertoire are motivated by the fact that some traditional animal models more closely related to humans present significant obstacles to researchers, including high cost, slow generation time, and complexity in measuring phenotypes. Increasingly, ethical issues are also preventing the use of our closest mammalian relatives as model organisms. In June 2013, the National Institutes of Health announced the retirement of chimpanzees in their research facilities following a report from the Institute of Medicine demonstrating that advances in biomedical research have enabled the use of alternative model organisms in studies traditionally utilizing chimpanzees [23]. These advances are a testament to the advent of new technologies that allow for the direct manipulation of a model organism’s genetics [24-26]; they also demonstrate the power of comparative genomic techniques in improving our understanding of animal genetics as a whole. Although there are logistical advantages to using simpler invertebrate animals as models, many questions remain regarding their suitability for human disease research. The choice of model organism for any given study has many contributing factors; primarily, a model organism must have analogous biological properties to the particular human condition of interest and must also be experimentally tractable. The extensive number of animal species with completed genome sequences provides a natural platform for a fresh analysis of the evolutionary distribution of disease genes for this purpose.

Previous studies on the origins of human disease genes found that the early animal lineages correspond to periods of rapid innovation for human disease genes [1-4,6], but these studies were conducted prior to the availability of whole-genome sequence in many of these lineages. Recent efforts to sequence the genomes of species representing the earliest-evolving animal phyla such as ctenophores (*Mnemiopsis leidyi* [27]), sponges (*Amphimedon queenslandica* [28])*,* placozoans (*Trichoplax adhaerens* [29]), and cnidarians (*Nematostella vectensis* [30]), as well as their closest non-animal relatives (*e.g.,* the unicellular filasterian *Capsaspora owczarzaki* and the unicellular choanoflagellates *Monosiga brevicollis* and *Salpingoeca rosetta*) [31], have increased our understanding of what shaped the evolution of multicellularity in animals and, by extension, what biological and physiological processes are universal to animals. Given these new data, we are now able to more thoroughly investigate the distribution of human disease genes across the Metazoa.

The utility of distantly related animal models for human disease research depends on the disease of interest and whether or not it is feasible to study in a particular organism. However, this distinction is not easy to make, as it requires determining the point in evolutionary time when a process related to human disease became functionally conserved. Often, this determination is made through identification of sequence orthologs which, under the assumptions of the “orthology-function conjecture” [32], would imply that sequence similarity across species is synonymous with functional similarity. However, there are caveats to the orthology-function conjecture; while it tends to hold true as a generalized, genome-scale approach for assigning function to newly sequenced genes, contradictory cases certainly arise [32]. Furthermore, determining whether a human Mendelian disease phenotype can be replicated in a distant ortholog may require consideration of characteristics beyond functional similarity, such as mutational effects and species-specific adaptation. Thus, while orthology is useful for identifying candidate genes of interest in a model organism, other context-specific conditions still need to be considered. Nevertheless, while the point in evolutionary time in which a disease gene emerged may not be equivalent to when it acquired its disease-related function, these two time points are likely correlated. Given this, the relative age of a disease gene class can be used as a criterion in selecting an appropriate species in which to study relevant underlying processes.

No study to date has analyzed the evolutionary distributions of specific *classes* of human disease genes. Rather, existing studies have focused on conservation at the levels of single genes, cancer-related genes [33], or on the superset of all human disease genes [3,6,34]. However, different disease classes exhibit diverse properties in gene interaction networks [2]; this suggests that, collectively, disease gene classes are not homogeneous. We hypothesized that disease gene classes also have heterogeneous evolutionary origins and pressures. A number of recent high-profile cases support this perceived heterogeneity; incongruities have been encountered between humans and closely related traditional animal models at both the genotypic and phenotypic levels for specific disease genes and classes, resulting in research findings that could not be translated into new treatments for human disease [14,24-26,35-40].

In this work, we have leveraged the vast amount of new whole-genome sequence data from a broad phylogenetic range of animals to analyze the evolutionary distributions of specific classes of human disease genes. We set out to accomplish three main goals: (1) to increase the resolution of evolutionary emergence patterns of human disease genes in animals, (2) to determine whether any specific disease classes show unique patterns of evolution, and (3) to perform an initial investigation into whether evolutionary metrics can help inform the process of selecting appropriate animal models (including “non-traditional” species) for studying the underlying genetics of specific human disease classes, citing a handful of recently reported cases where results generated in animal models could not be translated into humans.

## Results

### Evolutionary distribution of all human disease genes

The OMIM database [41] contains a manually curated set of human genes that are implicated in the causation of human genetic or genomic disorders. OMIM flags the highest confidence gene-disease associations as a “type 3” phenotype. These represent particular disease phenotypes in which the underlying molecular basis is known and has been mapped to a specific gene; at the time of this writing, this encompasses 3096 human genes. To analyze the distribution of these disease genes across animals and their closest relatives (unicellular filasterians and choanoflagellates), we overlaid the disease genes onto clusters of orthologous genes that were generated using a phylogenetically aware ortholog clustering algorithm with predicted protein sequences from 23 species (including human) whose genomes have been sequenced (see Methods and Additional file 1). 2727 of the OMIM disease genes were present in our clusters after filtering out genes that did not map to ENSEMBL proteins (270) or did not successfully cluster (99). Thus, using the clusters that contain at least one human OMIM disease gene, we obtain an evolutionary distribution of that disease gene based on the presence or absence of an ortholog in each of the 23 species.

**Figure 1:**
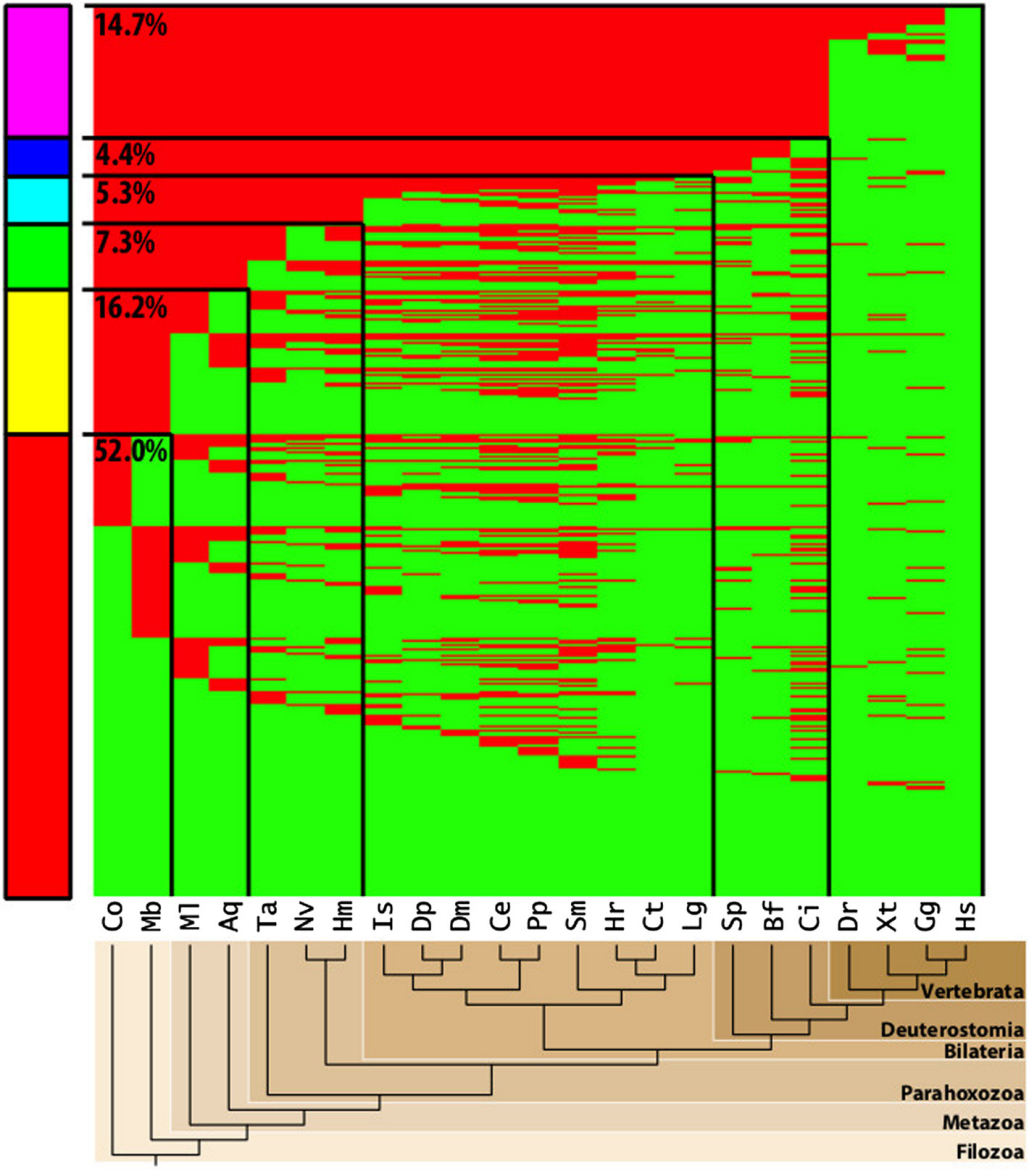
**Distribution of human disease gene orthologs.** Heat maps showing the presence (green) or absence (red) of an ortholog for a given human disease gene from OMIM (rows) within each species (columns). All 2727 human disease genes from OMIM are displayed. Major phylogenetic divergence events define the six phylostratigraphic bins indicated in the phylogenetic species tree. Rows are ordered such that disease genes first appearing in each phylostratigraphic bin (indicated by black lines) are clustered, with the corresponding percentage of the total for each cluster projected on the stacked bar on the left. Hs, *Homo sapiens*; Gg, *Gallus gallus*; Xt, *Xenopus tropicalis*; Dr, *Danio rerio*; Ci, *Ciona intestinalis*; Bf, *Branchiostoma floridae*; Sp, *Strongylocentrotus purpuratus*; Lg, *Lottia gigantea;* Ct, *Capitella teleta;* Hr, *Helobdella robusta;* Sm, *Schistosoma mansoni;* Pp, *Pristionchus pacificus;* Ce, *C. elegans*; Dm, *D. melanogaster*; Dp, *Daphnia pulex*; Is, *Ixodes scapularis*; Hm, *Hydra magnipapillata*; Nv, *N. vectensis*; Ta, *T. adhaerens*; Aq, *A. queenslandica*; Ml, *M. leidyi*; Mb, *M. brevicollis*; Co, *C. owczarzaki*.

We then analyzed the patterns in which these disease genes emerged within the Metazoa using phylostratification, a process by which genes are placed into major taxonomic groups (“phylostrata”) according to their inferred evolutionary emergence point [42]. We used the presence/absence distributions within clusters to bin disease genes into phylostrata based upon the lineage in which they first appeared in our analysis. Henceforth, we refer to each phylostratum by the most basal classification that it includes, although phylostrata are, in fact, hierarchical. Our analysis considers only species as distant as the Filozoa (*i.e.*, animals and their closest unicellular relatives), so this placement does not necessarily identify the emergence of a “founder gene” but, rather, characterizes the evolutionary patterns of gene families within and around animals. Figure 1 shows the full distribution of orthologs to the 2727 OMIM genes and the corresponding phylostratification, representing the evolutionary signature of all human disease genes.

In comparison to the superset of all human genes, we identify some of the same trends noted in previous studies [3,8,9]. Specifically, we observe that more than half of human disease genes are of ancient, pre-animal origins (52%), a number significantly larger than would be expected if disease genes were merely a random subset of all human genes (42.7%, p = 1.2 × 10^−25^ per one-tailed hypergeometric test; see Additional files 2 and 3). We also observe that surprisingly few human disease genes have origins within the vertebrates or later (14.7%), as compared to 25.9% of all human genes (p = 2.7 × 10^−50^ per one-tailed hypergeometric test). Notably, our inclusion of newly available whole-genome sequence data from the earliest animal lineages suggests that many human disease genes emerged with the first animals (16.2%). As with the pre-Metazoan set, this early animal gene set also represents a significantly larger proportion than would be expected based on all human genes (13.9%, p = 1.4 × 10^−4^ per one-tailed hypergeometric test). Overall, the complete phylostratigraphic distribution of human disease genes versus all human genes shows a significantly ancient skew (p = 2.2 × 10^−16^ per χ^2^ two-sample test; see Additional files 2 and 3).

### Species-specific human disease gene ortholog content

Given the ancient origins of the majority of the human disease gene set, we surveyed the total human disease gene ortholog content in the genomes of each species included in our analysis to assess their relative similarity to humans and, conversely, their propensity for gene loss and lineage-specific divergence. Even the animal phyla most distantly related to humans – ctenophores and sponges (represented by *Mnemiopsis* and *Amphimedon*) – contain orthologs to about half of all human disease genes (51.6% and 56.3%, respectively; see Figure 1 and Additional files 4 and 5). Immediately outside of the Metazoa, these percentages drop to 39.4% (for the choanoflagellate *Monosiga*) and 41.8% (for the filasterian *Capsaspora*). Our analysis identified a strikingly high number of human disease gene orthologs in the non-bilaterian cnidarian *Nematostella* (67.8%), consistent with previously reported findings that its genome is quite complex [9,19,30]. Comparatively, the average number of human disease gene orthologs present in the vertebrate (*D. rerio*, *X. tropicalis*, and *G. gallus*) and invertebrate (*S. purpuratus*, *B. floridae*, and *C. intestinalis*) deuterostomes that we studied was 93% and 73%, respectively.

We do not observe a steady increase in the number of orthologs identified relative to evolutionary divergence times from the human lineage, suggesting lineage-specific loss and divergence rates are not strictly dependent on evolutionary relation to humans. The nematode *C. elegans* contains orthologs to only 57.4% of human disease genes, a proportion smaller than *Nematostella* and hardly larger than the earliest branching metazoans, despite having diverged over 100 million years afterwards. This trend is also observed to a lesser degree in a few other protostomes, including the fruit fly *Drosophila*, whose percentage of observed disease gene orthologs is 64.2%. It is well known that the popular protostome models *C. elegans* and *D. melanogaster* have lost a number of genes important to human biology [9,30,43,44]. However, it is not immediately obvious whether most of these losses are shared amongst other protostomes, or whether they tend to be conserved in earlier-evolving phyla. Using our cluster analysis, we investigated how this particular set of disease genes is distributed with respect to the genomes of other animal species.

**Figure 2:**
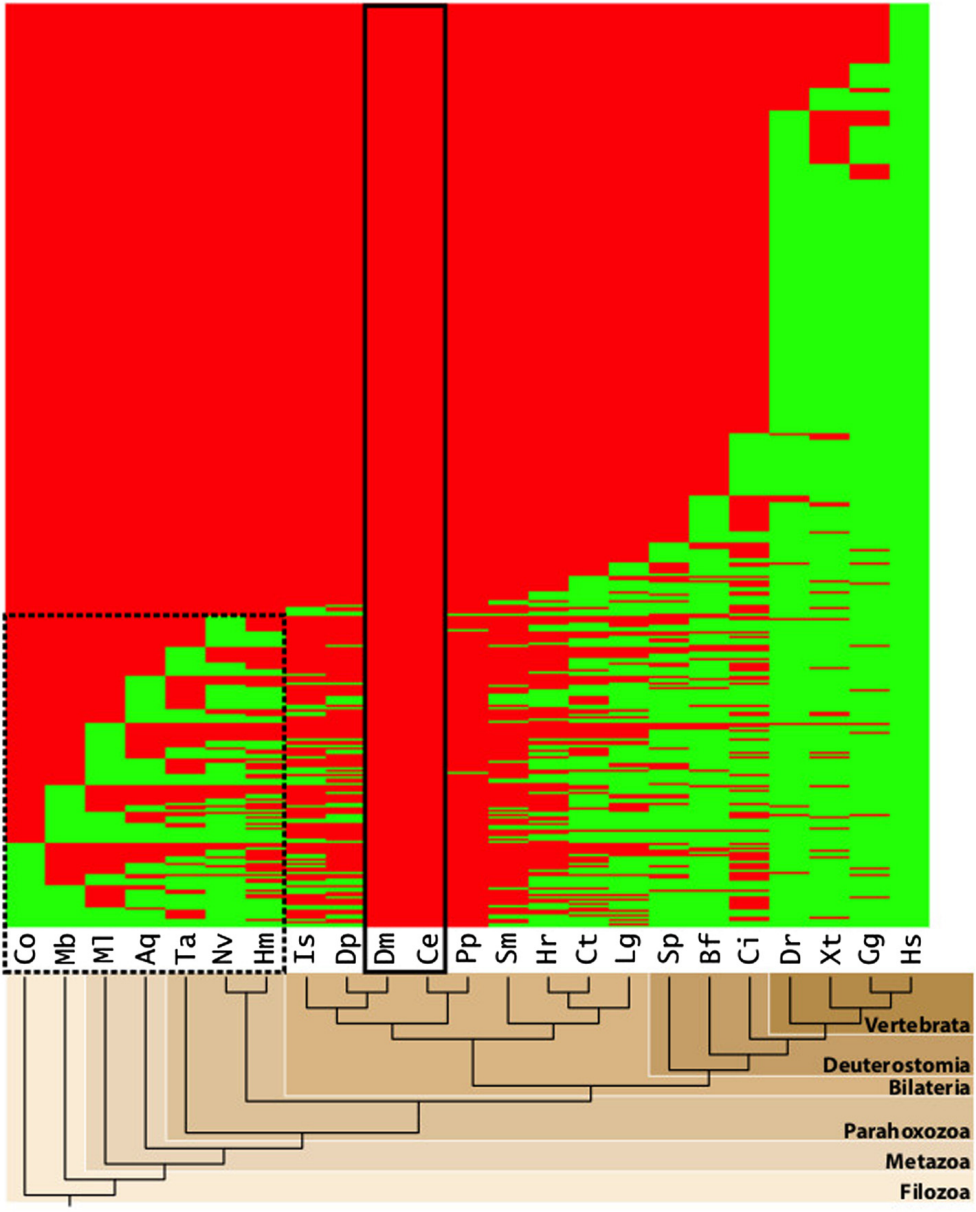
**Lineage-specific loss/divergence of human disease genes in** ***C. elegans*** **and** ***D. melanogaster*** **.** The subset of human disease genes (863 of 2727; see Figure 1) absent in both *D. melanogaster* and *C. elegans* (indicated by the solid black box), one-third of which are possible gene losses (292) due to their presence in a more distant phylostratum (dashed black box).

Figure 2 shows a heat map of OMIM genes in which an ortholog is absent from both *C. elegans* and *D. melanogaster* (863 out of 2727, or 31.6%). From these 863 missing genes, 292 are present in more distant non-bilaterian lineages, of which 223 are present in at least two such non-bilaterian species. This illustrates that nearly one-third of all human disease genes are absent (or highly derived) in both of these popular model organisms; in turn, one-third of these potentially represent gene losses and are not just the result of more recent evolutionary innovation. This analysis suggests that roughly 10% of all human disease genes could potentially be better-studied in selected non-bilaterian species than in either *C. elegans* or *D. melanogaster*, with *Nematostella* being an obvious candidate. For example, the breast cancer susceptibility gene *BRCA2* and the *BRCA1-*interacting protein BRIP1 are identified in every species studied except for the three protostomes *D. melanogaster*, *C. elegans*, and *P. pacificus*, with *BRCA2* additionally being absent in *L. gigantea*; *BRCA2* was previously identified to be well conserved in *Nematostella* [9]. While these remote animal species are less complex than humans, it is quite possible that studying the most distant forms of these genes would reveal insights into the most basic functions they evolved to perform and, by extension, their relationship to human disease. Additional statistics on all species studied are available in Additional files 4 and 5.

### Identification of disease classes with unique origins

On the one hand, our analysis has provided additional evidence that the majority of the human disease gene set has particularly ancient origins. We have expanded upon the notion that many of our most remote animal relatives contain large proportions of human disease gene orthologs. On the other hand, it is unlikely that all disease *classes* follow this same evolutionary model. We aimed to identify disease classes and disease-related biological processes that do not conform to the evolutionary profile exhibited by the superset of all disease genes. Currently, there is no “gold standard” disease gene annotation process that provides appreciable statistical power for analyzing these kinds of evolutionary profiles. While the OMIM database [41] does provide disease annotations for individual genes, we did not utilize these annotations because they are not guaranteed to be consistent across the database and are not widely standardized. Instead, we annotated the OMIM disease gene set with “level-1” and “level-2” functional classifications generated through the use of the Ingenuity Pathway Analysis (IPA) software suite (Ingenuity Systems®, http://ingenuity.com). IPA classifications are based on a curated, literature-derived knowledge base and have multiple levels of specificity; we chose to use the top two classification levels, corresponding to functional categories (level-1) and subcategories (level-2). In order to select for IPA classifications related to disease processes, we selected only those classifications whose occurrences were enriched in the disease gene set (Fisher’s exact test; p < 0.05, Benjamini-Hochberg-corrected). This method does not require that every assigned annotation reflect the same disease-causing mutation as reported in OMIM. Rather, a set of high-confidence annotations for diseases and disease-related processes is produced, for which a substantial set of known human disease genes play a role.

**Figure 3:**
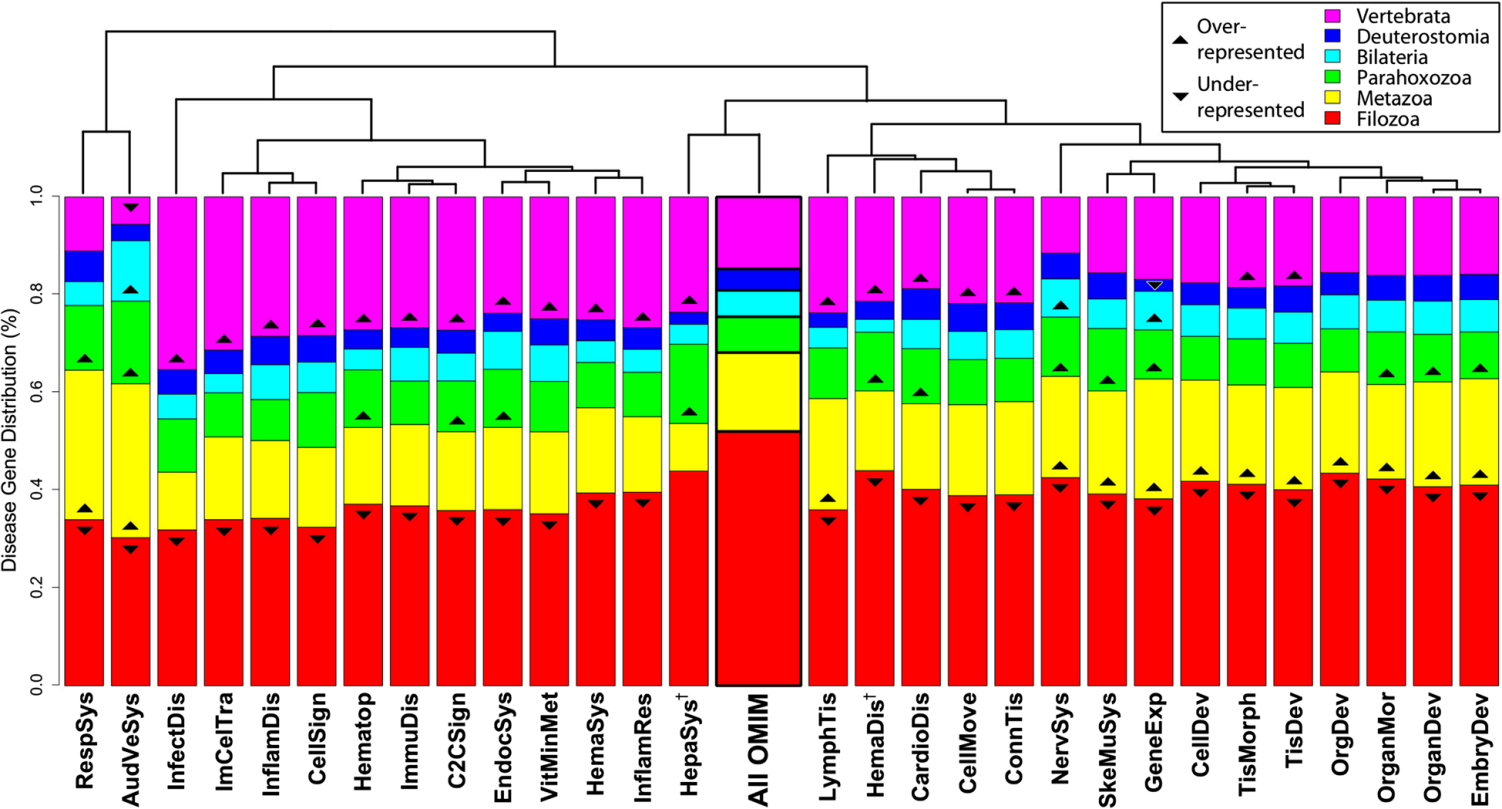
**Disease classifications with non-conforming evolutionary origins.** The 29 “level-1” disease-related annotations corresponding to genes with significantly deviating evolutionary distribution from the null model (“All OMIM”, see Figure 1 and Methods). Individual phylostratigraphic bins having over- or under-representation compared to the null model (Fisher’s exact test, 2 × 2 contingency table; p < 0.05) are indicated. Disease classes are hierarchically clustered by Euclidean distance-based similarity. RespSys (Respiratory System Development and Function), AudVeSys (Auditory and Vestibular System Development and Function), InfectDis (Infectious Disease), ImCelTra (Immune Cell Trafficking), InflamDis (Inflammatory Disease), CellSign (Cell Signaling), Hematop (Hematopoiesis), ImmuDis (Immunological Disease), C2CSign (Cell-to-Cell Signaling and Interaction), EndocSys (Endocrine System Disorders), VitMinMet (Vitamin and Mineral Metabolism), HemaSys (Hematological System Development and Function), InflamRes (Inflammatory Response), HepaSys (Hepatic System Disease), LymphTis (Lymphoid Tissue Structure and Development), HemaDis (Hematological Disease), CardioDis (Cardiovascular Disease), CellMove (Cellular Movement), ConnTis (Connective Tissue Disorders), NervSys (Nervous System Development and Function), SkeMuSys (Skeletal and Muscular System Development and Function), GeneExp (Gene Expression), CellDev (Cellular Development), TisMorph (Tissue Morphology), TisDev (Tissue Development), OrgDev (Organismal Development), OrganMor (Organ Morphology), OrganDev (Organ Development), EmbryDev (Embryonic Development). A dagger (†) denotes annotations not identified as significant with an alternative phylostratification method based on reciprocal BLASTP (see Methods).

For each enriched classification (and the corresponding subset of disease genes), we replicated our cluster-based phylostratigraphic analysis to compare the evolutionary distribution of the annotated genes to the distribution produced using all human disease genes from OMIM (see Figure 1). Disease-related subsets (referred to as “disease classes” throughout) displaying a statistically significant deviation in their complete phylostratigraphic distribution (Fisher’s exact test, 2 × 6 contingency table; p < 0.05, Bonferroni-corrected) were identified and analyzed. Of the 77 level-1 annotations considered, 48 (representing 62% of tested disease classes) were not found to significantly deviate from the null model (*i.e.,* the pattern observed for all OMIM genes as a whole). This included annotations for cancer, neurological diseases, and metabolic diseases (see Additional file 6). The results suggest that the majority of human diseases have very ancient origins, consistent with what we observed for the superset of all disease genes. However, the other 29 annotations (representing 38% of tested disease classes) were all under-represented in the Filozoa phylostratum (Figure 3), appearing more recently within the Metazoa than would be expected based on the null distribution. The same pattern is observed when considering level-2 annotations, in which 113 out of 500 classifications (22.6%) were found to significantly deviate from the null model, all exhibiting an under-representation in the Filozoa (see Additional files 6, 7, 8 and 9). Thus, there do not appear to be any disease classes with a significantly more ancient origin than the null distribution (*i.e.*, with over-representation of pre-metazoan genes), but there is a substantial set of disease classes that are characterized by sets of genes from more recent metazoan-specific innovations.

To ensure that these results were not an artifact of the IPA annotation process, which could potentially be biased towards more recently evolved genes, we compared the distributions of the set of disease genes that received no annotation (788) as well as the set that received at least one annotation (1939) to the null model of all OMIM genes. Both of these sets produce an evolutionary distribution almost identical to the superset of all OMIM genes (Fisher’s exact test, 2 × 6 contingency table; p = 0.93 and p = 0.99, respectively; see Additional file 2), suggesting that the IPA annotation process does not produce an evolutionary bias. Therefore, we can conclude that the 29 more recently emerging disease classes we have identified are exceptions to the ancient trend exhibited by the superset of *all* diseases, substantiating our concern that many diseases do not adhere to this overly generalized model of conservation.

### Novel signatures of disease class evolutionary origins

**Figure 4:**
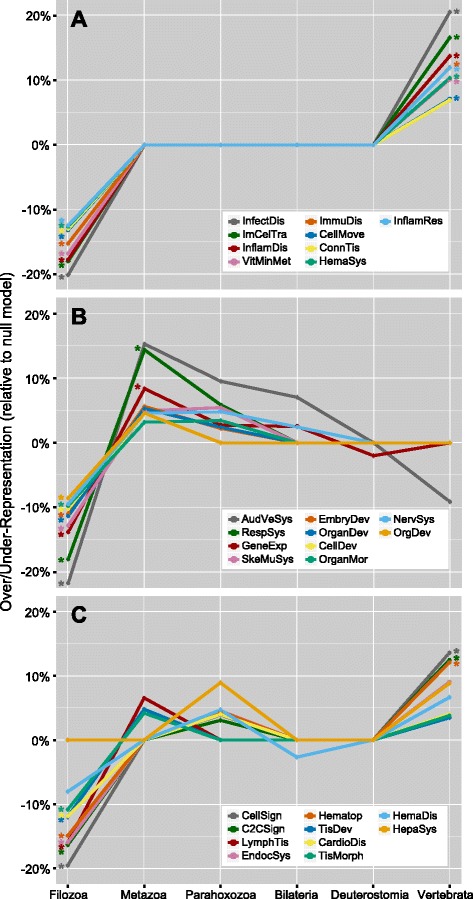
**Distinct evolutionary signatures of disease classes.** Comparison of the 29 “level-1” disease-related annotations identified as having a statistically significant evolutionary distribution (see Figure 3 and Methods), displayed relative to the distribution of the null model (all OMIM genes). Annotations are separated into signatures for **A)** vertebrate-specific, **B)** early metazoan, and **C)** multi-stage metazoan disease classifications. Only statistically significant over/under-representations of points within *individual* phylostratigraphic bins are plotted as non-zero (Fisher’s exact test, 2 × 2 contingency table; p < 0.05), corresponding to those denoted in Figure 3. Points marked with an asterisk (*) denote over/under-representations where p < 6.5 × 10^−4^ (p < 0.05, Bonferroni corrected for bin-specific comparisons).

Further analysis of the disease class-specific gene subsets revealed four recurring evolutionary patterns. The four patterns include the expected distribution displayed by all human disease genes and the majority of disease classes, with three of them being novel patterns that we refer to as “evolutionary signatures.” The first novel signature has genes evolving at expected rates between the emergence of metazoans and the deuterostomes, but appearing much more frequently than expected in the vertebrates and much less frequently prior to metazoans (Figure 4A). We term these the “vertebrate-specific” disease classes, which are the most recently evolved, and includes nine classifications such as Inflammatory Disease (InflamDis), Inflammatory Response (InflamRes) and Infectious Disease (InfectDis). For example, various sets of cytokines and their receptors were binned in the vertebrate lineage and linked to inflammatory diseases, including chemokines (CCLs), interleukins (ILs and ILRs), interferons (IFNGs and IFNGRs), and immunoglobulins (FCGRs). This group also includes some more ancient genes, such as sodium channel transporters (SCNs) and solute carriers (SLCs), perhaps representing ancient genes co-opted into the inflammatory response pathways at some point within vertebrate evolution.

The second novel signature (Figure 4B) is characterized by a set of disease classes that are under-represented outside of animals and over-represented in the early animal phyla (but not in the deuterostomes or vertebrates); we called these the “early metazoan” disease classes. This group of ten contains many developmental processes inherent to animals, including Embryonic Development (EmbryDev), Organ Development (OrganDev), and Organ Morphology (OrganMor), as well as processes related to the development and function of the Nervous System (NervSys), Skeletal and Muscular System (SkeMuSys), Respiratory System (RespSys), and the Auditory and Vestibular System (AudVeSys). While some of these systems may not have evolved in their entirety during the earliest stages of animal evolution, the data indicate that necessary components of these systems evolved within the Metazoa rather than prior to it, and more likely arose well before the evolution of the vertebrates.

The third signature (Figure 4C) seemingly represents an overlap between the “vertebrate-specific” and “early metazoan” signatures; we call these the “multi-stage metazoan” disease classes. This group of ten classes is distinguished by an under-representation outside the Metazoa and over-representations of lesser magnitude in both the non-bilaterian and vertebrate lineages. A number of these classifications include disease processes related to blood and the cardiovascular system, namely Cardiovascular Disease (CardioDis), Hematopoiesis (Hematop), Hematological Disease (HemaDis) and Hepatic System Disease (HepaSys). These signatures appear to have multi-modal distributions, indicating a more complex evolutionary history where different components of biological processes and diseases emerged at different periods in animal evolution, coinciding with major genomic innovation events.

### Differing rates of purifying selection act on disease classes of different age

Our phylostratigraphic analysis of disease gene age suggests that most disease genes evolved before or within the earliest vertebrate lineages. We estimate that >96% of the disease genes emerged before the divergence of zebrafish (*D. rerio*; see Figure 1). This is of particular importance given that there has been rapid and remarkable success in developing zebrafish into a standard animal model, especially after the sequencing of its genome [45]. However, recent cases of studies involving traditional model organisms that failed to produce results that can translate to humans [14,25,26,35-40] suggest that consideration of disease gene age alone (or the identification of an ortholog) may not be sufficient to rationalize the use of an organism as an appropriate model for studying human disease. Specifically, these reports have found inconsistencies relative to the human phenotype when studying inflammatory diseases in mice [35], certain immune responses in non-primates [14], and acute myocardial infarction drug candidates in dogs and rabbits [37]. Our analysis identified both Inflammatory Disease (InflamDis) and Immunological Disease (ImmuDis) as being grouped within the vertebrate-specific evolutionary signature, and various disorders related to blood and the cardiovascular system matching the multi-stage metazoan evolutionary signature [e.g., Cardiovascular Disease (CardioDis)]. As these two signatures are characterized by the over-representation of vertebrate-specific genes and under-representation of pre-metazoan genes, we can infer that these are the most recently evolved disease class signatures. Based on this inference, we hypothesize that a correlation exists between the relative genetic age of a disease and the evolutionary distance at which a particular model organism would be useful.

We posited that the presence of an unusual degree of purifying selection between a class of human disease genes and their orthologs in a model species may indicate potential problems for studying that particular disease class in that model organism. It has been shown previously that, in general, older genes evolve more slowly than younger ones [46]. However, it has also been shown that disease genes do not follow this trend; both younger and older disease genes appear to evolve slowly, at rates more similar to that of older genes [4]. Thus, we tested how different classes of disease genes behave by independently analyzing the selective pressures occurring on genes from each of the disease classes. We focused specifically on mammalian and primate species to see if a relationship exists between our identified signatures based on disease gene age and their evolutionary conservation within mammals. Sequence data were collected from nine well-characterized species: *Canis lupus familiaris* (dog), *Felis catus* (cat), *Rattus norvegicus* (rat), *Mus musculus* (mouse), *Oryctolagus cuniculus* (rabbit), *Otolemur garnetti* (bushbaby), *Callithrix jacchus* (marmoset), *Macaca mulatta* (macaque), and *Pan troglodytes* (chimpanzee).

**Figure 5:**
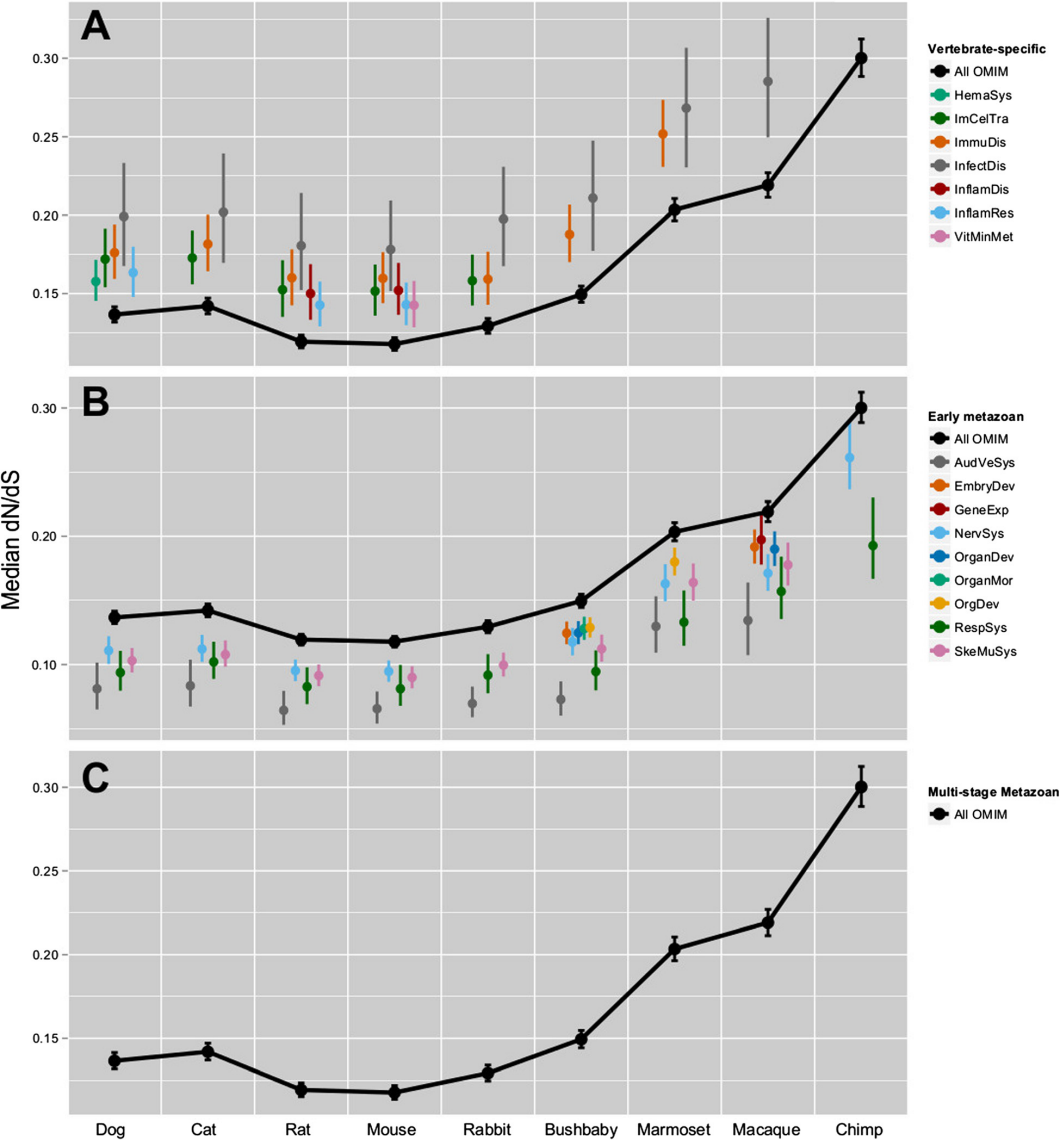
**Distinct evolutionary pressures on disease classes of different origins in popular mammalian models.** Comparison of the distribution of human disease gene dN/dS ratios for the 29 deviating disease classes, separated according to their age-related signature as defined in Figure 4: **A)** vertebrate-specific, **B)** early metazoan, and **C)** multi-stage metazoan classes, and compared across nine mammalian species relative to the expected distribution of all human disease genes from OMIM. Points indicate median dN/dS with 95% confidence intervals. Only statistically significant differences are displayed (Mann-Whitney U two-tailed test; p < 0.05, Bonferroni-corrected).

For each disease class, we analyzed the distribution of dN, dS, and dN/dS values for each disease gene within each of these mammalian species (see Methods). This analysis was restricted to mammals in order to maintain reliable dS rate estimates that can become saturated over larger evolutionary distances. We then compared these values to those calculated for the distribution of all 2727 human disease genes described above (Mann-Whitney U two-tailed test; p < 0.05, Bonferroni-corrected; see Additional file 10). This process enabled us to identify, for each mammalian model organism, disease classes having evolutionary rates that deviate significantly from the distribution observed for all human disease genes. To visualize and quantify positive or negative deviations, median values (with 95% confidence intervals) of the distributions for each metric were computed for each class of disease genes. Overall, the dS rates tended to follow the distribution observed for all human disease genes, but there were multiple cases of statistically significant deviations in dN rates. Henceforth, we only refer to the dN/dS ratio as it summarizes both statistics, noting that changes in dN/dS are predominately driven by changes in dN rates. Figure 5 shows the median dN/dS metric for each disease gene class found to significantly deviate from the distribution observed for all human disease genes in each mammalian species. The disease classes themselves have been grouped based on their age-based evolutionary signature identified via phylostratigraphic analysis (*i.e.*, vertebrate-specific, early metazoan, or multi-stage metazoan; see Figure 4).

Generally, the vertebrate-specific disease classes (Figures 4A and 5A) show weaker purifying selection than expected (*i.e.*, median dN/dS closer to 1 as compared to all human disease genes) in the mammalian species considered, whereas the early-metazoan disease classes (Figures 4B and 5B) show stronger purifying selection than expected (*i.e.*, median dN/dS closer to 0 as compared to all human disease genes). The magnitude of these trends varies for disease classes within each of the previously described evolutionary signatures. For example, the infectious disease class exhibits the largest over-representation of vertebrate-specific genes (Figure 4A); it also demonstrates the weakest degree of purifying selection in all mammalian species considered (Figure 5A). On the other end of the spectrum, vertebrate-specific disease classes that do not exhibit as large of an over-representation of vertebrate-specific genes (such as the connective tissue disorders and cellular movement classes) do not significantly deviate from the superset of all human disease genes by the dN/dS metric. In most cases, the deviations showing significantly weak purifying selection (*i.e.*, dN/dS closer to 1) more often occur in mammals of more distant relation to humans, supporting the logical conclusion that some disease classes are well conserved only in our closest animal relatives, but not necessarily in all mammals. We note that the human inflammatory disease genes tend to show weak purifying selection rates in mammals, with the deviation being statistically significant only in rat and mouse; this is consistent with our hypothesis that degree of purifying selection is related to appropriate model organism choices [35].

The multi-stage metazoan disease gene classes (Figures 4C and 5C) again appear to represent a combination of the vertebrate-specific and early-metazoan classes; some show slightly weaker purifying selection and some show slightly stronger purifying selection relative to all human disease genes, but the magnitudes of these differences are not considered statistically significant. The set of disease classes that were considered non-deviating from all human disease genes via phylostratification generally were also non-deviating in dN/dS values, with a handful (8 out of 48) being under stronger purifying selection than all human disease genes and having patterns similar to the early-metazoan cases (*e.g.*, “Behavior”, “Developmental Disorder”, “Neurological Disease”, and “Visual System Development and Function”; see Additional file 10).

To determine which genes are driving the deviation patterns exhibited in Figure 5A and B, we compared the dN/dS ratio distributions for all human disease genes between phylostrata. Only the vertebrate-specific set of human disease genes demonstrates a unique rate of purifying selection (significantly weaker than “older” genes), whereas all disease genes with pre-vertebrate origins are essentially indistinguishable from each other by this metric (see Methods and Additional file 11). This explains why disease classes with high proportions of vertebrate-specific genes are, overall, under weaker rates of purifying selection. It is important to note that we cannot rule out the possibility that difficulties in identifying distant homologs of rapidly evolving genes could lead us to believe that they are younger. However, the stronger rates of purifying selection observed in the early-metazoan disease classes seem to be more context-specific, reflecting unique evolutionary pressures acting on these specific biological processes opposed to a universal pattern for all disease genes of early metazoan origins. This suggests that some of the human disease-related processes that evolved with multicellularity in the earliest animal lineages are potentially under stronger evolutionary constraints than those with unicellular origins.

## Discussion

Recent additions of non-bilaterian animal species with whole-genome sequence data available motivated us to analyze the evolutionary origins of human disease genes, with a particular focus on these early periods of animal evolution. One goal of this analysis was to determine the potential utility of these species in modeling the genetics underlying specific classes of human disease. With these genomic data in hand, our results corroborate previous findings that the majority of human disease genes are of particularly ancient origins, having many more genes of pre-animal origin than would be expected if disease genes were a random subset of all human genes [3-5]. Through our analysis of additional early metazoan genomes, we find that there is also a significant over-representation of early metazoan genes in the human disease gene set, suggesting that the ancient skew of human disease gene origins extends into the early animal lineages as well (see Additional files 2 and 3).

More importantly, we have shown that not every class of human disease genes has ancient origins. Rather, a subset of disease classes (38% of those examined) show significantly more recent origins than the superset of all human disease genes, with many first appearing more frequently within the Metazoa and Vertebrata. We identified three novel evolutionary signatures from this set, all representing disease classes with over-representations of metazoan-specific genes: the vertebrate-specific, early metazoan, and multi-stage metazoan. Furthermore, we show that these patterns, based on relative genetic age of a human disease class, are correlated with the observed rates of evolutionary selective pressures (dN/dS ratios). These two observations are made on different evolutionary time scales, having age measured on a pre-mammalian scale and evolutionary pressures measured within mammals and primates. Specifically, we have shown that human disease genes with vertebrate-specific origins tend to be under weaker levels of purifying selection within mammals than human disease genes of pre-vertebrate origins. This result contradicts findings that younger disease genes are evolving particularly slowly compared to non-disease genes; they appear to more closely mimic the rates observed among all genes [4,46]. As a result, classes of human disease that contain many vertebrate-specific genes are unlikely to be as highly conserved in certain mammalian models as disease classes of more ancient origins. Thus our results may provide some insight regarding a handful of recent experimental findings addressing whether mice are poor models of inflammatory diseases [35] and if certain immune response genes can only be studied in primates or humans [14], for example. Our evolutionary profiling studies identified both inflammatory diseases and immunological diseases as having large proportions of vertebrate-specific genes that are under weaker-than-expected purifying selection, particularly in mice and rats for inflammatory diseases and all species beyond old world monkeys for immunological diseases.

Conversely, we also demonstrated that certain disease classes with many genes of early metazoan origins are under particularly strong rates of purifying selection within mammalian lineages. This suggests that some functional groups of human disease genes that arose at the base of the Metazoa (and the biological processes that they are responsible for) have distinct evolutionary pressures. As a group, they appear to be more highly conserved than both older and younger human disease genes. While the evolutionary rates were not computed beyond the eutherian mammals (due to issues of mutational saturation in dS values), we speculate that this trend continues to deeper branches of the metazoan tree of life. To enable the investigation of subsets of disease genes not considered in our analysis, we have provided the complete phylostratigraphic distribution of our disease gene set and corresponding dN/dS ratios in Additional files 6 and 10, respectively.

In practice, if the distribution of dN/dS ratios for a set of human disease genes with orthologs in a particular species is in fact correlated with the degree to which the underlying disease process can be modeled, then our results indicate that disease classes with over-representations of vertebrate-specific genes may be harder to mimic outside of our closest animal relatives. However, the opposite trend is observed for disease classes with over-representations of genes found in the earliest metazoan lineages, which appear to be under particularly strict selective pressures; these disease genes may be possible to study in our more distantly related animal relatives. In total, our phylostratigraphic analysis of individual disease classifications has demonstrated that the majority of disease classes (62% of those examined) do have ancient origins, consistent with the distribution of all human disease genes. This collective evolutionary model tends to have large proportions of genes that pre-date animals and are well conserved within mammals. Thus, this majority set of disease classes may also be promising candidates to study in a more diverse set of animal species.

Our results imply that there may be utility in studying disease genes that have primarily pre-vertebrate origins in non-traditional animal models, especially in the case of genes known to be lost or highly derived in popular protostome models. From our analysis, we estimate that as many as 10% of all human disease genes are absent or highly derived in both *C. elegans* and *D. melanogaster*, but have an ortholog in at least one more-distantly related species. These species generally have fast regeneration times, short life cycles, are inexpensive to culture, and can teach us about the evolutionary context of conserved disease genes and the most basic functions they evolved to perform. Nonetheless, it is important to factor in the experimental tractability of these species, most of which have not been developed into standard model organisms. By endorsing their value to human disease research through surveys such as this one, it is hoped that the biomedical community will give serious consideration to expanding the standard repertoire of model organisms to include non-bilaterian animals. While non-bilaterians such as sea anemones and ctenophores may not seem tractable for human gene modeling, efforts are currently underway to increase their utility as “emerging model organisms” [18,20-22].

## Conclusions

Taken together, the patterns we have identified highlight the need for a wider evolutionary perspective to be considered when selecting appropriate model organisms for studying a particular human disease or disorder. Our results indicate that a one-to-one comparison of the human disease gene complement in a target model organism is insufficient to rationalize its use as a good model. Rather, analysis of the evolutionary history corresponding to the entire disease process being studied, as well as establishing the system-wide context in which it plays a part, can be decidedly more informative. We caution against over-generalizing and approaching all human disease genes as uniformly evolving collections of genes. This further emphasizes the need to make model organism choices on a case-by-case basis in consideration of evolutionary origins, experimental tractability, and many other context-specific factors.

Future efforts to extend and refine our analyses could theoretically produce methods that could direct an investigator to a set of model species that would be well-suited to studying a particular human disease gene or disease class. That said, there are many obstacles that make this difficult to achieve at the present time. First, the development of a standardized database or ontology for annotating disease genes would be necessary to enable the comparison of disease phenotypes in greater depth and breadth. Second, to more precisely decipher the human disease gene content and tractability of emerging animal models, additional sequence data from species in the more sparsely represented sections of the animal phylogenetic tree will be required. The majority of existing data from these regions are EST traces that suffer from low gene coverage [47,48]; efforts to expand these genomic data with high-throughput transcriptome sequencing may provide an alternative to whole-genome sequencing for the purpose of mapping gene content [49]. Third, as new genomic data continue to be generated for these distant species, our work could be extended with a method that more comprehensively characterizes gene family relationships beyond orthology, such as the methods used for the EnsemblCompara GeneTrees [50] or other methods that define gene age according to more dynamic properties [51]. To our knowledge, these methods have yet to be applied with the addition of newly sequenced genomes, especially those from non-bilaterian animal lineages. The use of a phylogenetic gene tree-based method would also enable the estimation of dN values beyond mammals, which appear to be the driving force of the dN/dS ratio deviations we have identified. Thus, despite dS value mutational saturation issues, it may be possible to perform an analysis of selective evolutionary rates of human disease genes over a larger evolutionary distance by considering dN rate estimates alone. Finally, in order to more thoroughly investigate cases where certain animal models would be inappropriate, there needs to be a platform by which negative results can be reported in the literature. With the addition of these hypothetical data and the improvement of methodologies for defining disease gene family evolution on a genome-wide scale, it may be possible to develop comparative genomic tools to pinpoint suitable animal models in a context-specific fashion.

## Methods

### Phylostratigraphic analysis with clusters of orthologous genes

Clusters of genes with putative orthology in humans and 22 other species were generated using sequence similarity based on BLAST and relative position in a predetermined phylogenetic species tree (see Figure 1). We assigned bit scores to hits between each pair of genes by summing those for initial BLASTP high-scoring segments (HSPs) found on the same pair of genes, in consistent order, and overlapping less than 5% (with bit scores penalized proportional to the amount of overlap, computed as the larger of overlap_fraction * HSP_score/HSP_length for the two HSPs). We determined orthologous sets of genes at each tree node in two steps. First, if a set or gene from one child of the node was in a mutual best hit-relation with a set or gene from the other child, they were combined into a new set. Second, following this initial merge step, we then considered all hits within the node’s subtree and between the subtree and all outgroup genes in descending order of bit score (in either source-target gene direction). A better hit to an outgroup gene blocked any further merging of a gene or set (until another tree node was visited), while a hit between two sets or genes within the subtree, neither previously blocked, resulted in these being merged into a new set. This orthology computation was based on that described and implemented for the genome sequence of *Nematostella* [30] with further refinement of the blocking rules. Merging of species tree nodes (and of the underlying sets of orthologous genes, where in a mutual-best-hit or unblocked-hit relationship) continues iteratively until the root node of the species tree is reached. Additional information on the method’s implementation is provided in Additional file 12.

The resulting clusters represent families of orthologous genes, and the distribution of genes within a cluster provides a picture of the presence/absence of a gene family within the representative set of animals (and their closest outgroups). The subset of clusters containing human OMIM genes was used for further phylostratigraphic analysis. The phylostratification produced by this method is more conservative than that of an unbiased, complete BLAST database query to identify gene orthologs by similarity threshold, such as the methods used in previous phylostratigraphic studies [3], because the method adheres to a discrete phylogenetic hierarchy, uses a scoring metric that takes the length of sequence matches into account, and is duplication-aware to the extent of distinguishing orthologs and out-paralogs (in-paralogs would be interpreted as two distinct ancestral genes at any particular tree node, but can become in-paralogous during subsequent merges of parental tree nodes). Therefore, it aims to increase specificity at the cost of sensitivity and provides higher-confidence orthologous gene relationships than those based simply on sequence similarity below a given threshold. This method inherently employs a Dollo-type parsimony model of gene evolution by assuming that gene families evolved (or duplicated) once in a single common ancestor. In order to qualitatively assess and contextualize the clusters from our analysis, we compared the clustering results of a few gene families with known human disease gene members to their previously reported phylogenetic relationships. Our results, which are in agreement with these previously reported phylogenetic relationships, show that our clusters are able to detect a wide range of phylogenetically meaningful relationships (see Additional files 13 and 14).

### Evaluation of evolutionary selection rates of disease gene classes

We downloaded dN and dS evolutionary rates for all human RefSeq genes from BioMart, which are pre-computed for mammalian orthologs in the EnsemblCompara GeneTrees [50]; dS values become saturated over larger evolutionary distances, restricting the species we considered to a subset of eutherian mammals. We assigned dN, dS, and dN/dS values to each human gene ortholog from each of the nine mammalian species considered, selecting only the top ortholog based on orthology confidence, sequence identity, and minimum dN/dS ratio (in order). Of 19170 human genes considered, between 85.0% (rabbit) and 91.8% (chimpanzee) were assigned dN and dS values. Of the 2727 disease genes used in our phylostratigraphic analysis, between 91.6% (rabbit) and 98.3% (mouse) were assigned, and within disease class subsets, between 88.1% and 100% were assigned, with a median value of 96.3%.

To determine whether disease genes from any particular phylostratum had unique relative rates of selection, we compared the dN/dS rate distributions of genes from each phylostratum, restricted to the superset of all human disease genes with dN/dS ratios identified in every selected mammalian species (2103 out of 2727 genes). Within each mammalian species, dN/dS rate distributions were compared between all pairs of phylostrata (Mann-Whitney U two-tailed test; p < 0.05, Bonferroni-corrected). Only the vertebrate-specific set shows any significant difference in dN/dS distribution compared to the gene sets in other phylostrata, and this difference is significant in every comparison except for, within chimpanzee, vertebrate-specific genes versus genes in the Parahoxozoa and Bilateria phylostrata, respectively (see Additional file 11).

### Reproducibility of disease gene phylostratification using reciprocal-best BLASTP searches

In order to assess the robustness of the phylostratification produced by the clusters and the reproducibility of the resulting evolutionary signatures of disease classes presented in Figures 3 and 4, we performed a second phylostratigraphic analysis using an alternative method. In this case, all OMIM genes with an NCBI RefSeq protein counterpart (2874 total, including all 2727 from the ortholog clusters) were queried against predicted protein sets of each species independently with a reciprocal BLASTP search. Thus, a given OMIM gene was identified as a reciprocal best BLASTP hit (RBH) to a non-human gene within a given species if the OMIM gene and candidate non-human gene were both best BLASTP hits to the other, having E-values of less than 1 × 10^−3^ in both cases. Thus, the RBH relationships between human genes and the genes of a non-human species are one-to-one and consider only OMIM genes, opposed to the relationships defined by the ortholog clusters that are many-to-many and include all human genes. The RBH method is, therefore, even more specific than the ortholog clustering method, but also less sensitive, and should not be expected to produce an identical phylostratification. Nonetheless, we use this method to demonstrate that the results of our analysis are reproducible and not dependent upon the phylostratigraphic method. The results of the RBH method are presented in Additional file 6.

We identified the overlap between the two methods and found that 23,374 OMIM gene orthologs were also identified as RBHs (excluding *D. pulex*, *C. intestinalis*, and *X. tropicalis* due to problems arising from differences in protein sequence identifiers; see Additional files 4 and 5). This represents 70.2% of all OMIM orthologs identified and 78.1% of all OMIM RBHs identified. Overall, we found the two methods to be in relatively strong agreement despite their stated differences, but this highlights the fact that identifying homologs of human genes from diverse animal species is not an exact science. In particular, it is confounded by the abundance of gene families that arose via duplication(s) of a common ancestor and the interpretation as to which evolutionary event (*i.e.*, the emergence of the founder gene, its duplication, or some other intermediate event) is the most relevant. The most robust method would require running phylogenetic trees on each OMIM gene, which is not currently a tractable approach on a large, multiple gene family scale because each tree is sensitive to the selection of a suitable set of gene sequences (including outgroups) and must be interpreted manually. Other methods have considered treating emergence and most-recent duplication events independently [6,50], but this complicates the ability to study evolutionary distributions of large sets of genes and has not yet been applied to newly sequenced genomes.

In addition to the reproducibility of individual ortholog or RBH assignments for OMIM genes, we also evaluated the reproducibility of the phylostratification (*i.e.*, the identification of the most distant ortholog) produced by each method and the subsequent selection of significantly deviating disease processes. These results are presented in Additional file 15. We found that 1696 of the 2727 OMIM genes that were considered in both methods were placed in the same phylostratum, representing 62.2% reproducibility. When allowing for an assignment error to an immediately adjacent bin (e.g., a gene identified as first appearing in the Metazoa by one method, but in either the Parahoxozoa or Filozoa in the other), the reproducibility increases to 2173 (79.7%); thus, nearly half of the inconsistencies between binning methods are still close in relative evolutionary distance. Another main source of discrepancy lies in the fact that the ortholog clustering method tends to produce a more ancient skew relative to the RBH method, likely because RBHs are more stringent in their similarity metric and more prone to false negatives stemming from distantly diverging sequence. Thus, an ortholog is identified in the Filozoa for 52% of the OMIM genes, whereas only 46.2% have a RBH.

Despite the noted differences in the phylostratigraphic methods, however, the selection of disease annotations that significantly differ from the null (“All OMIM”) model is highly reproducible across methods. Figure 3 identifies 29 significantly skewed disease distributions, with 27 of these significantly skewed using the RBH method as well. Therefore, the ortholog method identifies only two annotations not reproduced by the RBH method and, likewise, the RBH method identifies 11 uniquely (see Additional file 6). These unique results generally have less dramatic deviation from the null distribution than those that are reproduced. When comparing the distributions for reproduced annotations, the overall distributions are expectedly not identical. However, the most statistically significant over- and under-representations are well maintained between the two methods. We notice a number of annotations that interchange between the multi-stage metazoan group (Figure 4C) and either the vertebrate-specific (Figure 4A) or early metazoan (Figure 4B) group between phylostratigraphic methods, but no cases exist where a disease annotation was placed in the vertebrate-specific group by one method and the early metazoan group by the other. Therefore, some of the marginal over-representations identified in one method may not be identified as significant in the other, but we found no cases of major disagreement. Finally, we compared the distributions for each annotation (both non-deviating and significantly deviating) across phylostratigraphic methods and found that while the two distributions for each matching annotation are not identical, they are more similar than expected based on all pairs of across-method distribution comparisons (calculated using χ^2^ two-sample test statistics adjusted for equal degrees of freedom, one-tailed t-test of means where N = 76 and 5927 defined comparisons, p = 0.0086).

### Estimation of sampling errors from choice of taxa

The evolutionary distributions we have compared are dependent upon the sample of species used and their grouping into phylostrata. Our analysis is limited by the few species with whole-genome sequence available in distant animal lineages. While the goal of our analysis was not to identify founder genes, we nonetheless estimated what percentage of genes have origins much deeper than the Filozoa, and more specifically, how many of those were considered in our analysis to be Metazoa-specific. For example, if a disease gene has more ancient origin than the species we have studied but was lost in both *M. brevicollis* and *C. owcarzaki*, then we cannot conclude whether a gene binned in the Metazoa in fact has Metazoa-specific origins, or if it was simply lost in the Filozoan species we included. To estimate the occurrence of these events, we performed an independent RBH comparison of the human disease gene (OMIM) set versus the genome of the yeast *S. cerevisiae*, yielding a set of 676 genes out of 2874 (23.5%). This was used as a representative set of genes that could be considered to have pre-metazoan origins with relatively high confidence. We then compared the phylostratification of this gene set by each phylostratigraphic method to see how often these yeast genes were placed in the most basal phylostratum (Filozoa), indicating likely pre-metazoan origins.

For the ortholog clustering method, 655 of the 676 yeast homologs were included in our clusters. 572 of these were binned in the Filozoa, suggesting the other 83 are found only in our metazoan genomes, indicating potential loss in the filozoans we studied. This represents about 3% of the 2727 clustered genes: 51 were instead binned in the Metazoa, eight in the Parahoxozoa, five in the Bilateria, four in the Deuterostomia, and 15 in the Vertebrata. For the RBH method of phylostratification, 47 of the 676 yeast homologs were not binned in the Filozoa, representing about 1.6% of the 2874 genes with RBHs. 25 of these were instead binned in the Metazoa, 12 in the Parahoxozoa, four in the Bilateria, one in the Deuterostomia, and five in the Vertebrata. These data suggest that despite not looking beyond the Filozoa, very few genes with likely pre-metazoan origins were considered Metazoa-specific in our analyses.

## Availability of supporting data

The data sets supporting the results of this article are included within the article and its additional files.

## Additional files

Additional file 1:
**Species included in the phylostratigraphic analysis and the accompanying predicted proteome sources.**
Additional file 2:
**Number of genes binned in each phylostratum for gene sets including all human genes, human disease genes (OMIM), or human disease genes that did or did not receive at least one annotation from IPA.**
Additional file 3:
**Comparison of the evolutionary distribution of the human disease gene subset (orange) versus all human genes (y=0; data not shown).** χ^2^ two-sample test, p = 2.2 × 10^−16^. Numbers of genes binned into individual phylostrata were further compared, showing over-representations in the Filozoa and Metazoa phylostrata and under-representations in the Deuterostomia and Vertebrata phylostrata (hypergeometric test; ***p < 1.0 × 10^−20^, **p < 0.001, *p < 0.01). Additional file 4:
**Number of OMIM genes identified in each species by each phylostratigraphic method.** [An asterisk (*) denotes taxa deemed unreliable due to mismapped sequence identifiers]. Additional file 5:
**Number of human disease gene (OMIM) orthologs and RBHs identified in each species studied, with overlap between the two indicated.**
Additional file 6:
**Results of phylostratigraphic analyses and disease gene annotations.**
Additional file 7:
**Vertebrate-specific level-2 disease annotations.**
Additional file 8:
**Early metazoan level-2 disease annotations.**
Additional file 9:
**Multi-stage metazoan level-2 disease annotations.**
Additional file 10:
**Disease gene dN, dS, and dN/dS values in each mammalian species considered.**
Additional file 11:
**Median dN/dS ratios for all disease genes in each phylostratum for each mammalian species considered.**
Additional file 12:
**Pseudocode describing the ortholog clustering algorithm used for phylostratification of human disease genes.**
Additional file 13:
**Clusters of human disease genes that are known members of multi-gene families.** (A) Apolipoproteins, (B) Caspase enzymes, and (C) components of the TGF-β signaling pathway. Additional file 14:
**Qualitative assessment of ortholog clusters [** 52-63 **].**
Additional file 15:
**Number of genes binned in each phylostratum by ortholog clusters and RBHs.**
