# Supplementary figures and images for: Evolutionary profiling reveals the heterogeneous origins of classes of human disease genes: implications for modeling disease genetics in animals
Source: BMC Evol Biol. 2014 Oct 4;14:212. doi: 10.1186/s12862-014-0212-1 (PMC4219131; doi:10.1186/s12862-014-0212-1)

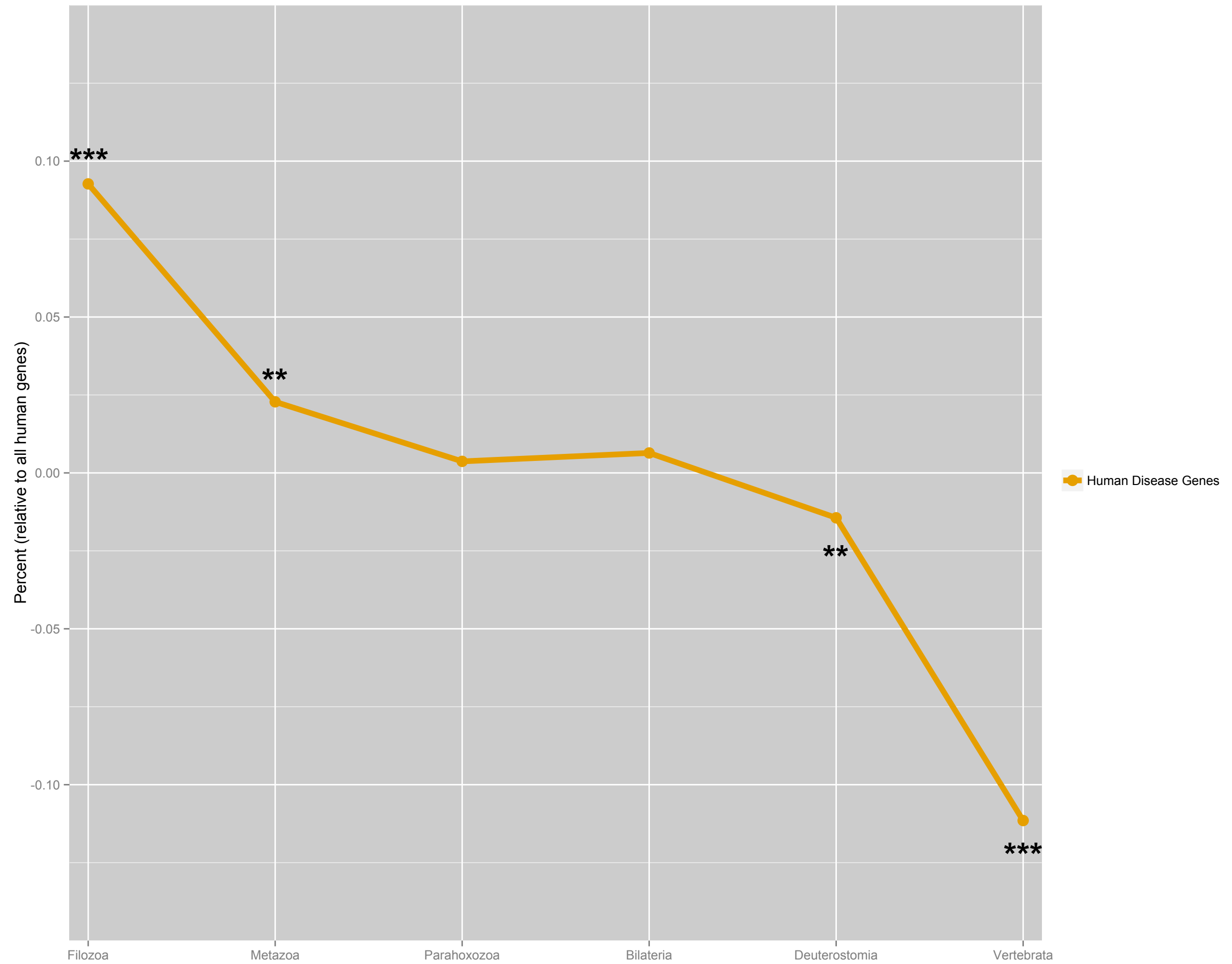

Supplement: Additional file 3: — Comparison of the evolutionary distribution of the human disease gene subset (orange) versus all human genes (y=0; data not shown). χ2 two-sample test, p = 2.2 × 10^−16^. Numbers of genes binned into individual phylostrata were further compared, showing over-representations in the Filozoa and Metazoa phylostrata and under-representations in the Deuterostomia and Vertebrata phylostrata (hypergeometric test; ***p < 1.0 × 10^−20^, **p < 0.001, *p < 0.01). [file 12862_2014_212_MOESM3_ESM.pdf]

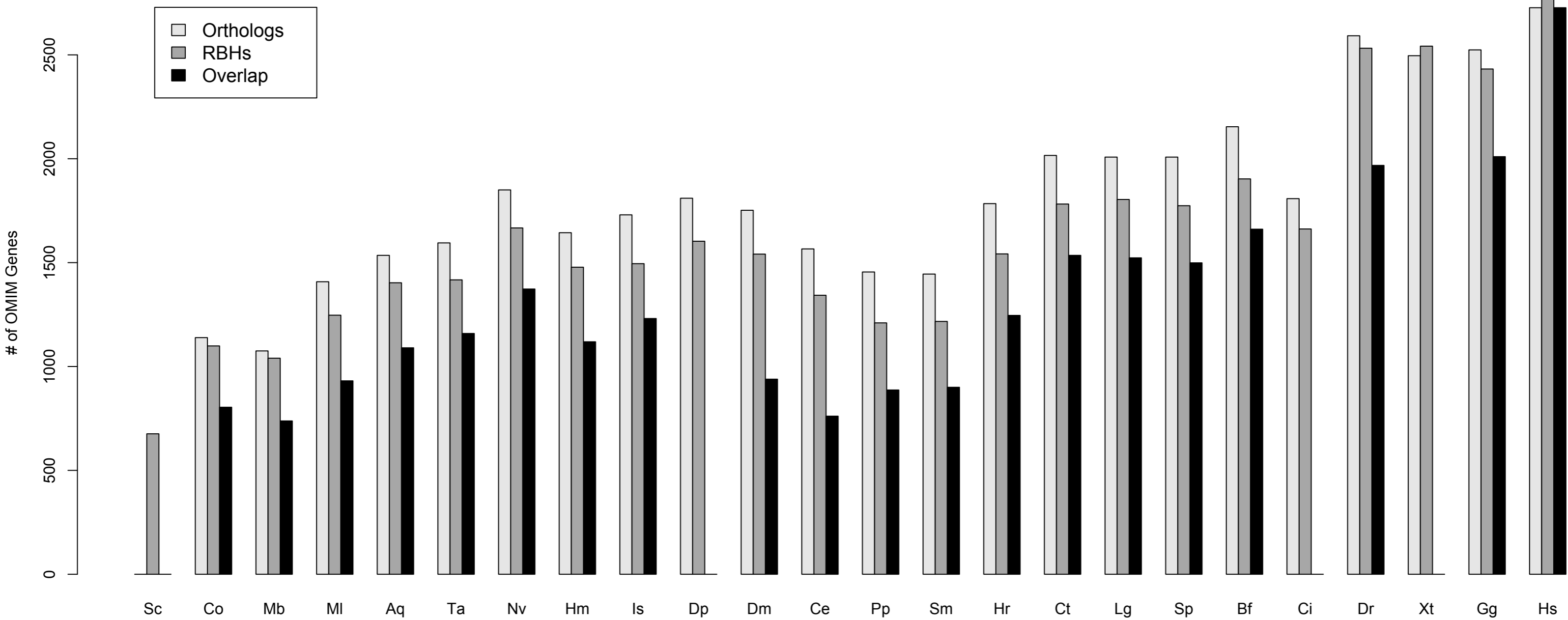

Supplement: Additional file 5: — Number of human disease gene (OMIM) orthologs and RBHs identified in each species studied, with overlap between the two indicated. [file 12862_2014_212_MOESM5_ESM.pdf]

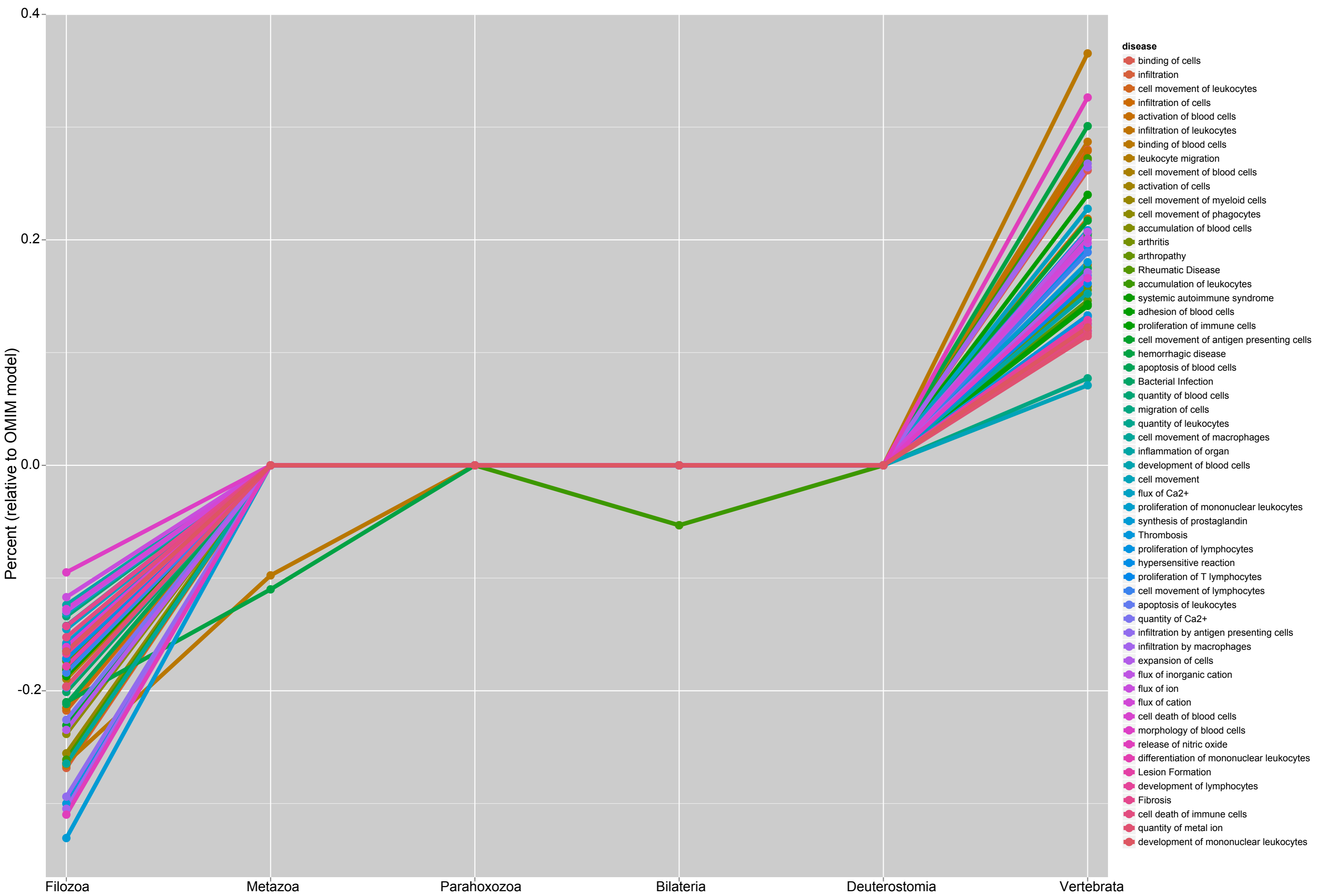

Supplement: Additional file 7: — Vertebrate-specific level-2 disease annotations. [file 12862_2014_212_MOESM7_ESM.pdf]

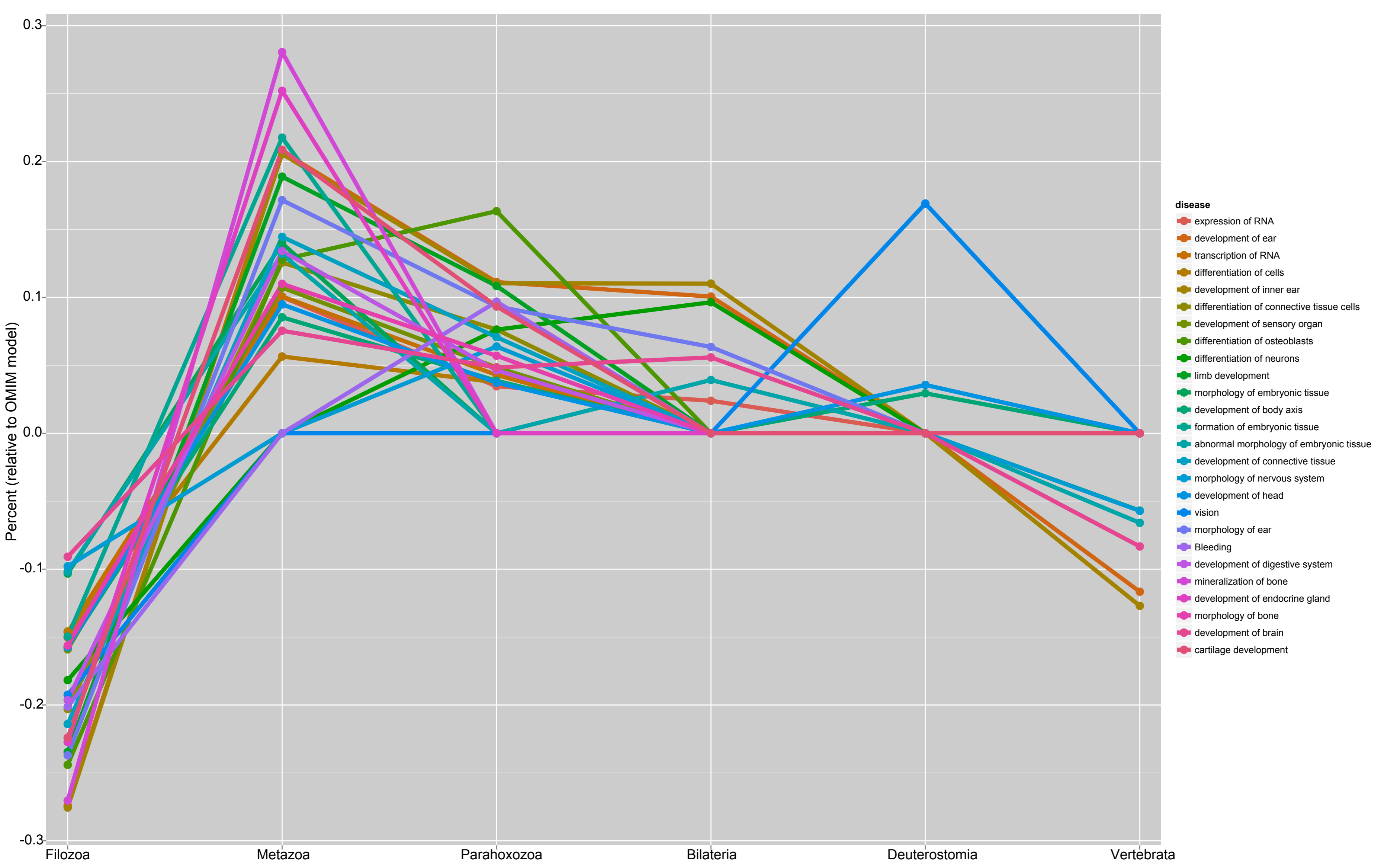

Supplement: Additional file 8: — Early metazoan level-2 disease annotations. [file 12862_2014_212_MOESM8_ESM.pdf]

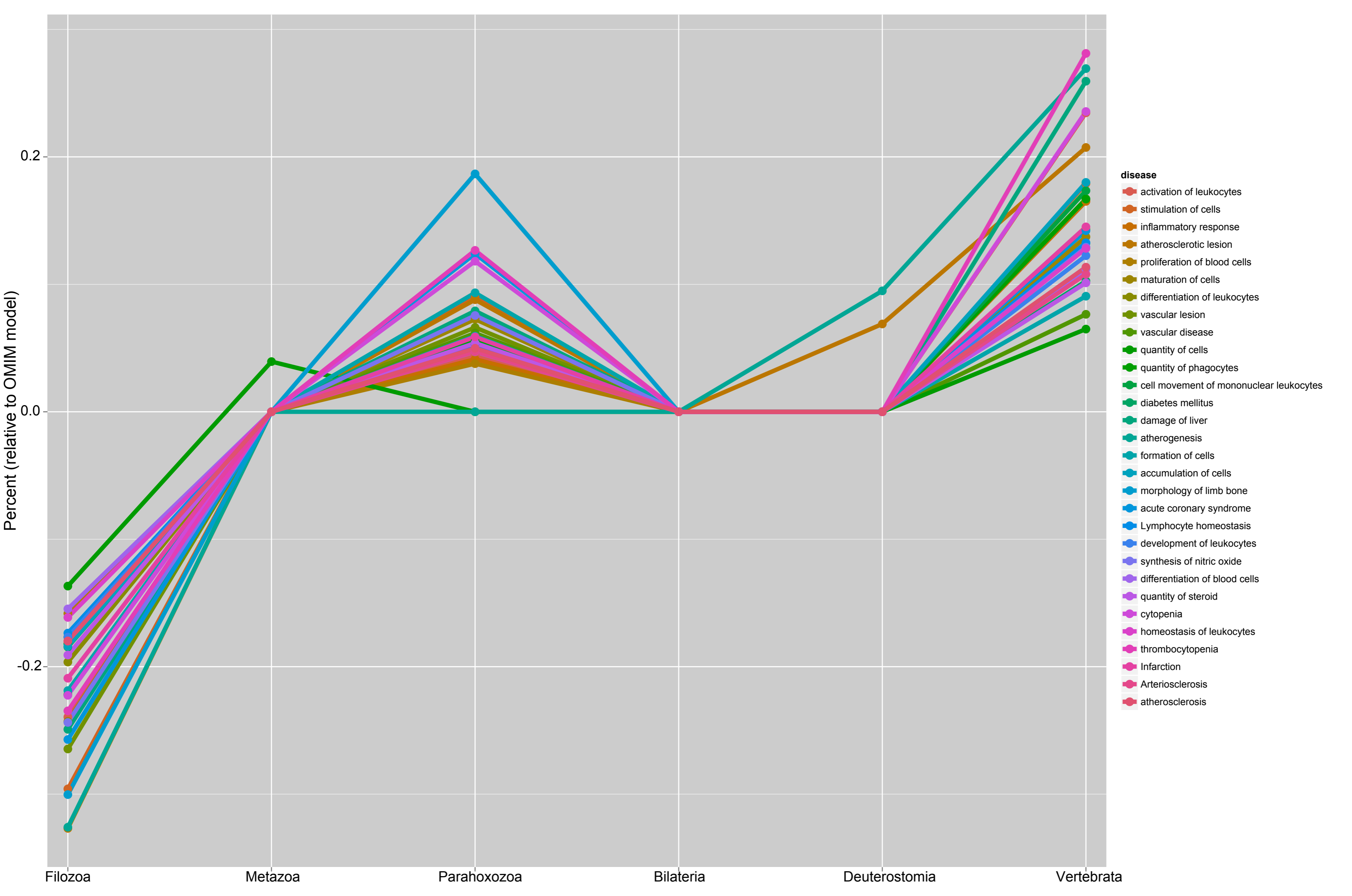

Supplement: Additional file 9: — Multi-stage metazoan level-2 disease annotations. [file 12862_2014_212_MOESM9_ESM.pdf]

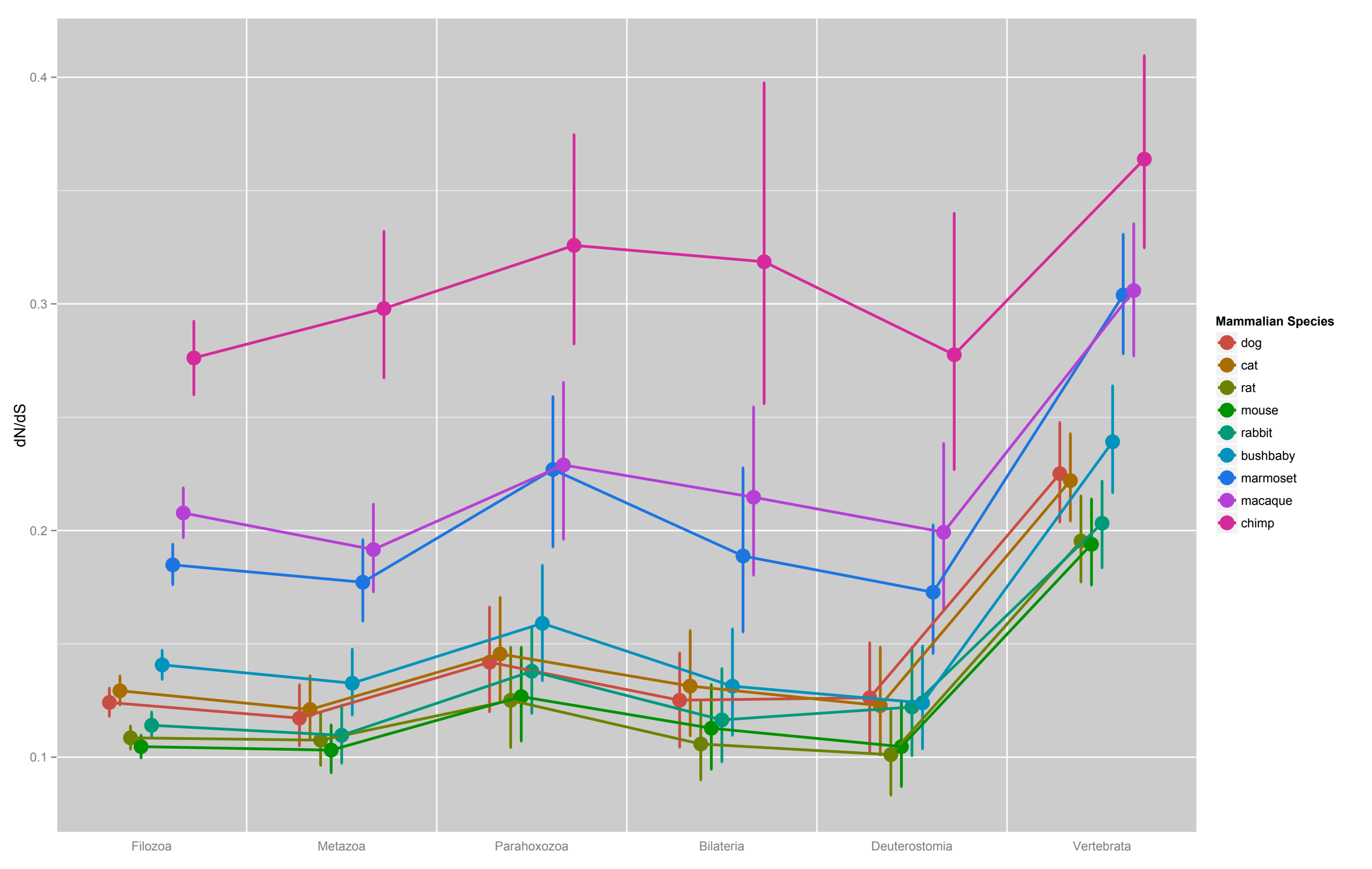

Supplement: Additional file 11: — Median dN/dS ratios for all disease genes in each phylostratum for each mammalian species considered. [file 12862_2014_212_MOESM11_ESM.pdf]
