# Supplementary material for: Evolutionary profiling reveals the heterogeneous origins of classes of human disease genes: implications for modeling disease genetics in animals
Source: BMC Evol Biol. 2014 Oct 4;14:212. doi: 10.1186/s12862-014-0212-1 (PMC4219131; doi:10.1186/s12862-014-0212-1)
Supplement: Additional file 12: — Pseudocode describing the ortholog clustering algorithm used for phylostratification of human disease genes. [file 12862_2014_212_MOESM12_ESM.rtf]

Orthology analysis for proteomes over a tree of speciesCo-authors in the Putnam lab at Rice University analyzed gene orthology for this paper using the same method they employed for [64]. Our method develops gene families based on sequence similarity and a phylogenetic tree of species, like its parent method developed at JGI for [30, 65], and is thus a sibling of the PhIGs approach [66].This tree-based hierarchical strategy produces at each node of the species tree a set of gene families. Just as each leaf node represents a current species and each internal node a putative common ancestor, so each initial gene family is a single gene and each internal-node gene family represents a putative ancestral gene that later diversified by speciation and duplication.The species tree is constrained to be a binary tree, and the set of gene families at an internal node is built by merging some gene families of its children. The nesting of gene families over all nodes therefore constitutes a gene tree — but not a binary one, as we save only the final result of multiple merges within a node (so that one saved gene family at a node may be the parent of more than two families from its child nodes).Inputs:	species phylogenetic tree		(child, parent) edges connecting species nodes			At the root:				Root node that is parent to the full-binary subtree				Global outgroup organisms directly below the root node					(These will always be in the outgroup, but organisms of					the “active tree” below will be in the ingroup when they					are in the subtree of a node being processed.)			A full binary subtree, the “active tree”, over which gene family					results will be computed				L leaf nodes				L-1 internal nodes	a proteome for each leaf species, comprised of a set of peptide sequences,		one or more for each gene of interest (selecting the longest one or more		if there are multiple peptides for the same gene locus)Outputs:	for each species-tree leaf or internal node		a saved_families set, partitioning the peptides into disjoint gene families		- the relevant peptides are from this organism (if a leaf node)			or in organisms in the subtree rooted here (if an internal node)	over all leaf and internal nodes, the saved_families sets implicitly define a 			forest of peptide phylogenetic trees (but not binary ones)Steps:Weights for all gene-pair edges from BLASTP bit scores	# Note that bit scores are used because they are easily summed for multiple 	# sub-hits (“high scoring pairs” or HSPs) and they do not depend on database	# size, so need not be adjusted when using a different species tree.	Label peptides by standard numeric identifiers efficient reference in later stages		- identify representative/longest peptide(s) for gene loci with multiple			transcripts; ignore others	for each leaf or outgroup proteome A		for each leaf or outgroup proteome B			- BLASTP the peptide sequence files of A vs. B (even if A = B)			foreach pair of peptides P in A and Q in B				- create a graph of HSPs (“high-scoring pairs”) between P and Q				- make graph nodes of the top 200 or fewer HSPs (by bit score)				- create an edge between each pair of HSP nodes with consistent					order of the aligned regions in P and in Q					- except if the aligned regions overlap by five percent or more					- weighted by the bit score of the destination-node HSP					- penalized by the fraction of overlap times the score				- create special START and STOP nodes connected to every HSP node,					with each edge from START to an HSP weighted by that HSP’s bit score				- the result is a directed acyclic graph with every node lying on at					least one path from START to STOP				- find the highest-weight path from START to STOP					if that path has total weight >= 30							and the span of path HSPs is >= 5 percent in both sequences							and the base coverage of path HSPs is >= 5 percent in both sequences						make that total weight the saved weight (bit score) between P and Q				- note that bit scores are log-odds scores, meaningful to sum			save all weights computed thus as directed gene edge weights for A vs. B			# running BLASTP in both orders is found to be more robust	for each pair of leaf or outgroup proteomes A and B		with numeric identifier of A less than that for B		# so that we handle A vs. B and B vs. A on the same iteration		foreach pair of peptides P in A and Q in B			take maximum of directed weights in A vs. B and B vs. A as undirected P-Q weight		save the gene-pair edge information sorted by descending weight as edges(A, B)Treewalk	Each gene family is represented by an arbitrary representative peptide, 	with other peptides pointing directly or indirectly to the representative	according to Tarjan’s UNION-FIND algorithm. At each node, there are two 	steps: making a list of initial merges (for leaf-node genes at the same 	genomic locus, or for internal-node merges on mutual best hits between	left and right children), then performing those merges plus additional	merges on all unblocked merges.	# note that initially, all internal nodes have unprocessed children, 	# but no leaf nodes do	Unprocessed = nodes of the “active tree”	# in_families are the initial families computed at each node,	# 	while Unprocessed is nonempty		remove a node A, having zero children in Unprocessed, from Unprocessed			# there is at least one, and this ensures bottom-up tree traversal		if A is a leaf node			initial_merges = { for each multi-peptide genomic locus, merge 				all peptides with a representative longest peptide }			# note that non-longest peptides are not included in the BLASTP			# runs, and therefore enter gene families at this point only		else A is an internal node			initial_merges = MBH_merges(left_child(A), right_child(A))		saved_families(A).Merge_initial_and_unblocked(initial_merges, 												left_child(A), right_child(A))		# saved_families(A) is the result for this nodeFamilies_Set.Merge(P, Q)	Union(Find(P), Find(Q))MBH_merges(L, R)	L_families = saved_families(L)	R_families = saved_families(R)	for each gene family X in L_families and R_families		best_hit[X]    = None		best_weight[X] = 0	for each gene-pair edge (l, r) between L and R		# that is, in edges(x, y) or edges(y, x) for x in L and y in R		family_L = L_families.Find(l)		family_R = R_families.Find(r)		if (l, r).weight > best_weight[family_L]			best_hit[family_L]    = family_R			best_weight(family_L] = (l, r).weight		else if (l, r).weight == best_weight[family_L]				and best_hit[family_L] != family_R			best_hit[family_L] = None		if (l, r).weight > best_weight[family_R]			best_hit[family_R]    = family_L			best_weight[family_R] = (l, r).weight		else if (l, r).weight == best_weight[family_R]				and best_hit[family_R] != family_L			best_hit[family_L] = None	merges_list = () # empty list	for each family_L in L_families		if best_hit[best_hit[family_L]] == L			merges_list.append(family_L, best_hit[family_L])Families_Set.Merge_initial_and_unblocked(merges_list, L, R)	initialize families set from saved_families(L) + saved_families(R)		# disjoint from each other at first	for each family f		blocked_score[f] = 0	for each merge (p, q) in merges_list		Merge(p, q)	construct a file stream F from a merge_sort of sorted files 		(requiring space proportional to N = the number of files, and 		 extract_max_edge() taking time proportional to log(N))		giving edge records in descending order of weight		where the previously sorted files are 			L.prev_blocked, R.prev_blocked,			all edges(x, y) for one of x & in L and the other in R,			all edges(w, z) for one of w & z in L+R the other 				in outgroup(L+R) # (i.e., anywhere else in the tree)	# Edges are used to merge unless they are blocked by an out group	# edge of greater or equal score. To handle ties, pending merges	# are collected until we’ve progressed to lower-scoring edges	# without seeing a block.	pending_list = ()	pending_score = 0	# for clarity of presentation, treat F as though it has	# a null edge of zero score appended at the end	for each gene-pair edge e from F		if e.weight < pending_score			blocked_list = process(pending_list)			print blocked_list			pending_score = 0		if e.weight < 30 # usual minimum score			return		if (e.source in L+R and e.target in outgroup(L+R))			t_family = Find(e.target)			blocked_score[t_family] = e.weight if < e.weight		else if (e.target in L+R and e.source in outgroup(L+R))			s_family = Find(e.source)			blocked_score[s_family] = e.weight if < e.weight		else			# an ingroup edge			pending_score = e.weight			pending_list.append(e)Families_Set.process(pending:edge_list) returns blocked:edge_list 	for each edge e in pending		x = Find(e.source)		y = Find(e.target)		# high blocking scores propagate to any family we try to 		# merge with		if (blocked_score[x] < blocked_score[y])			blocked_score[x] = blocked_score[y]		else			blocked_score[y] = blocked_score[x]		if (blocked_score[x] < e.score)			# because processing in descending order, should only happen			# if blocked_score[x] is zero			Union(x, y)		else # blocked			# x and y won’t be merged now; need save only the first blocked 			# edge between them (the highest-scoring merge attempt)			if (x and y not already blocked)				blocked.append(e)	return blockedMethod References[64] Simakov O, Marletaz F, Cho S-J, Edsinger-Gonzales E, Havlak P, Hellsten U, Kuo D-H, Larsson T, Lv J, Arendt D, et al.: Insights into bilaterian evolution from three spiralian genomes. Nature 2013, 493:526–531.[65] Putnam NH, Butts T, Ferrier DEK, Furlong RF, Hellsten U, Kawashima T, Robinson-Rechavi M, Shoguchi E, Terry A, Yu J-K, et al.: The amphioxus genome and the evolution of the chordate karyotype. Nature 2008, 453:1064–1071.[66] Dehal P, Boore JL: Two rounds of whole genome duplication in the ancestral vertebrate. PLoS Biology 2005, 3:e314.
