# Supplementary material for: Evolutionary profiling reveals the heterogeneous origins of classes of human disease genes: implications for modeling disease genetics in animals
Source: BMC Evol Biol. 2014 Oct 4;14:212. doi: 10.1186/s12862-014-0212-1 (PMC4219131; doi:10.1186/s12862-014-0212-1)
Supplement: Additional file 13: — Clusters of human disease genes that are known members of multi-gene families. (A) Apolipoproteins, (B) Caspase enzymes, and (C) components of the TGF-β signaling pathway. [file 12862_2014_212_MOESM13_ESM.pdf]

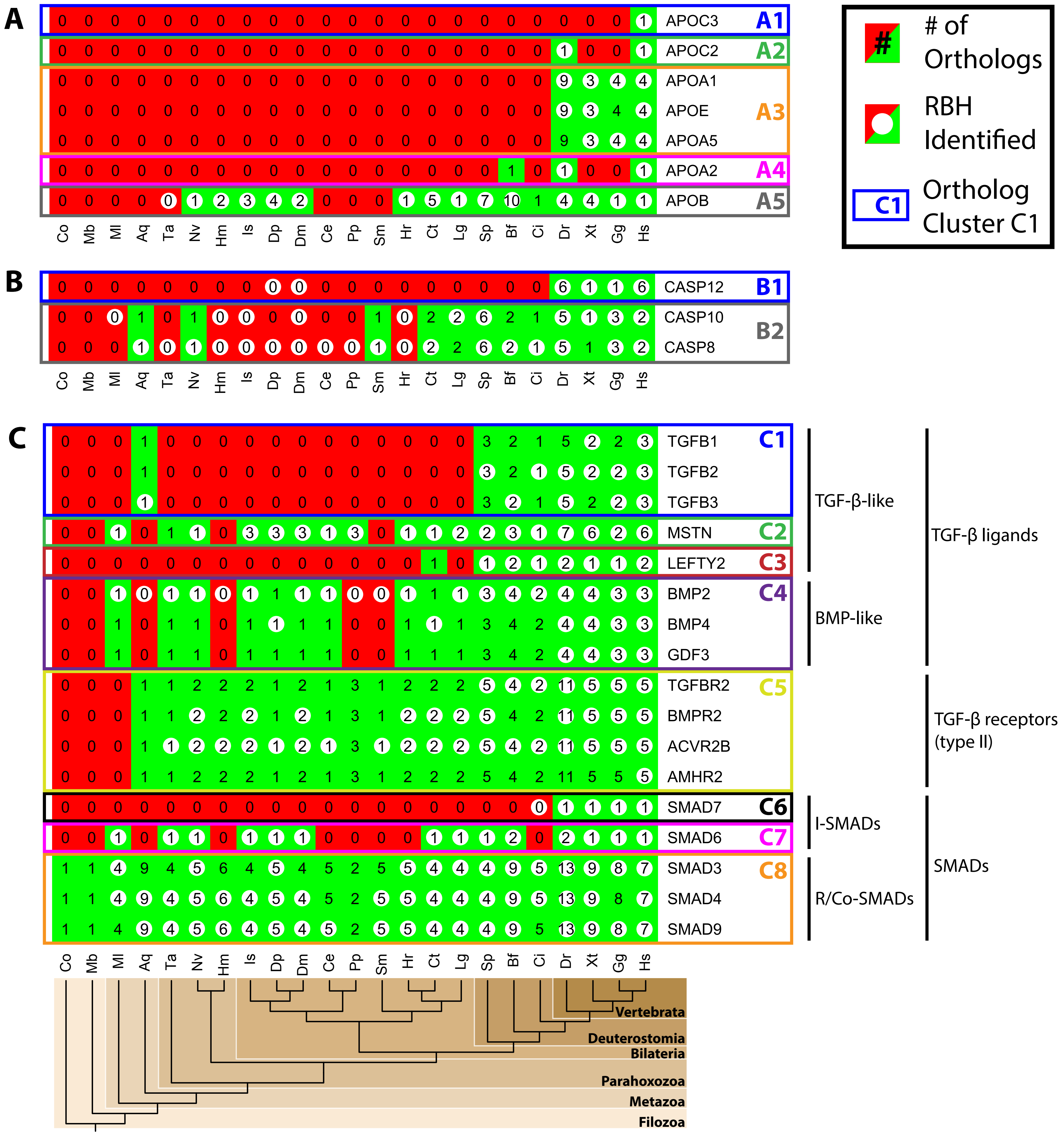
A
APOC3
A1
APOC2
A2
APOA1
APOE
APOA5
A3
APOA2
A4
APOB
A5
Co Mb Ml Aq Ta Nv Hm Is Dp Dm Ce Pp Sm Hr Ct Lg Sp Bf Ci Dr Xt Gg Hs
# of Orthologs
RBH Identified
C1
Ortholog Cluster C1
B
CASP12
B1
CASP10
CASP8
B2
Co Mb Ml Aq Ta Nv Hm Is Dp Dm Ce Pp Sm Hr Ct Lg Sp Bf Ci Dr Xt Gg Hs
C
TGFB1
TGFB2
TGFB3
C1
MSTN
C2
LEFTY2
C3
BMP2
BMP4
GDF3
C4
TGFBR2
BMPR2
ACVR2B
AMHR2
C5
SMAD7
C6
SMAD6
C7
SMAD3
SMAD4
SMAD9
C8
TGF-β-like
BMP-like
TGF-β ligands
TGF-β receptors (type II)
I-SMADs
R/Co-SMADs
SMADs
Co Mb Ml Aq Ta Nv Hm Is Dp Dm Ce Pp Sm Hr Ct Lg Sp Bf Ci Dr Xt Gg Hs
Vertebrata
Deuterostomia
Bilateria
Parahoxozoa
Metazoa
Filozoa
