# Supplementary material for: Evolutionary profiling reveals the heterogeneous origins of classes of human disease genes: implications for modeling disease genetics in animals
Source: BMC Evol Biol. 2014 Oct 4;14:212. doi: 10.1186/s12862-014-0212-1 (PMC4219131; doi:10.1186/s12862-014-0212-1)
Supplement: Additional file 14: — Qualitative assessment of ortholog clusters [ 52-63 ]. [file 12862_2014_212_MOESM14_ESM.docx]

In order to qualitatively assess our orthologous gene clusters of human disease genes, we compared our clusters to well-studied human disease genes that are members of multi-gene families with known phylogenetic relationships. Specifically, we considered gene families whose evolutionary origins have been studied with extensive phylogenetic analyses, including gene family-specific phylogenetic trees that include an evolutionarily appropriate set of species and gene candidates. In practice, these are the types of in-depth, gene family-specific analyses that should be done on a human disease gene of interest prior to selecting a specific model organism. Thus, we present this analysis as a proof-of-concept in regard to the validity of our clusters and their improvement over one-directional BLAST searches, while demonstrating the contextual aspects that need to be further considered for individual disease genes.

*Apolipoproteins*

Additional file 13, Panel A shows a set of apolipoprotein human disease genes and their corresponding orthologous gene clusters. The *APOB* gene and the other *APO* genes are distinct from one another; *APOB* apolipoproteins form low-density lipoprotein whereas the others form high-density lipoprotein. Furthermore, *APOB* has been shown to share a common ancestor with the *Vtg* gene, which appears to have arisen ~700 million years ago in the early Parahoxozoa [52, 53], consistent with our Cluster A5. We note that Cluster A5 does not contain an ortholog in *T. adhaerens* (but does contain a RBH to *APOB*), likely due to the difference in domain structures between the *Vtg* ortholog identified in *T. adhaerens* and the rest of the gene family, including *N. vectensis* orthologs [52]. Thus Cluster A5 is consistent with the reported phylogenetic gene tree for *APOB* and *Vtg* [52] by grouping the orthologs in *N. vectensis* and *D. melanogaster* with the human *APOB*, but excluding the genes that are more *Vtg*-like in *T. adhaerens* and *C. elegans*.

However, the other apolipoprotein genes arose from an unrelated evolutionary event, likely pre-dating tetrapods and teleosts in the earliest vertebrates [53, 54]. This appears to have represented the ancestral form of the human *APOA*, *APOC*, and *APOE* genes (and possibly others). However, subsequent local or full gene duplications have diversified the gene family, with the *APOE*, *APOA1*, and *APOA5* human disease genes (and the non-disease gene *APOA4*; data not shown) all having at least six more duplications of 11 or 22 codons than the *APOA2* and *APOC* genes [55, 56]. This evolutionary history corresponds logically with our Clusters A1-A4.

*Caspases*

Caspase (cysteinyl aspartate proteinase) enzymes have multiple roles in metazoan programmed cell death (apoptosis) and inflammation, which are central to many human diseases. Specifically, *CASP8*, *CASP10* and *CASP12* were amongst our OMIM set of human disease genes, and we show their corresponding clusters in Additional file 13, Panel B. These three caspases group in two clusters; *CASP12* forms Cluster B1 with five other human non-disease genes (including *CASP1*, *CASP4*, *CASP5*, *CARD16* and *CARD17*; data not shown), while *CASP8* and *CASP10* form Cluster B2. These relationships are consistent with previous phylogenetic analyses of caspases, where *CASP8* and *CASP10* form their own subfamily of initiator caspases with a death effector domain (DED) in place of the caspase recruitment domain (CARD) [57-59]; an ortholog to this subfamily has previously been identified in the sponge *A. queenslandica*, but not outside of the Metazoa [28]. Furthermore, the absence of an ortholog (but presence of an RBH) in *D. melanogaster* and *C. elegans* is expected, as those species have similar, but lineage-specific caspases (e.g. DREDD, DECAY, DRICE, and DRONC) [57, 58]. *CASP12*, on the other hand, is an inflammatory caspase more closely related to *CASP1*, *CASP4*, *CASP5*, and *CASP11* than to *CASP8* and *CASP10* [59, 60].

*TGF-β pathway components*

Transforming growth factor β (TGF-β) signaling is an important developmental pathway found only in metazoans [61-63]. In our disease gene set, we find multiple components of the pathway, including multiple ligands, receptors, and intracellular SMAD proteins. Within the ligands, we find disease genes from both the TGF-β-like and BMP-like groups, including *TGFB1-3*, *Myostatin*, *LEFTY2*, *BMP2/4*, and *GDF3* (Additional file 13, Panel C). The TGF-β-like ligands form Clusters C1-C3, whereas all of the BMP-like ligands cluster together in Cluster C4.

Cluster C1 represents the TGF-β *sensu stricto* ligands (*TGFB1-3*). These ligands were originally thought to exist only in vertebrates, but the identification of homologs in *A. queenslandica* and *M. leidyi*, as well as similar ligands in other species, suggested they emerged at the base of the Metazoa [61-63]. In this cluster, orthologs are found in all Deuterostomes and also the poriferan *A. queenslancia*. This demonstrates the ability of our orthologous gene-clustering algorithm to identify distant orthologs in cases where many species, from the Parahoxozoa up until Deuterostomia, have lost the underlying gene. However, our clusters only identify the *A. queenslandica* ortholog and not the *M. leidyi* ortholog, demonstrating that largely diverged sequences are harder to classify. It has been previously demonstrated that the *M. leidyi* TGF-β *sensu stricto* ligand is the least homologous member of the ligand family [62, 63]. We note that although the *A. queenslandica* ortholog is most similar to the *TGFB3* human counterpart (based on the RBH results), the clustering suggests that the ancestral form duplicated in the Deuterostomes to form the larger class of three TGF-β *sensu stricto* ligands seen in humans.

Cluster C2 contains the human disease gene myostatin (*MSTN*) as well as five other human non-disease genes representing inhibins (*INHBA/B/C/E*) and *GDF11* (data not shown). Cluster C3 contains the human disease gene *LEFTY2* as well as *LEFTY1*, a non-disease gene (data not shown). Taken together, the grouping of Clusters C1-C3 is meaningful from both a sequence-similarity perspective as well as a phylogenetic origins perspective. While these various classes of TGF-β are all distinct, they likely have a common ancestor dating back to the earliest metazoans. However, the Lefty ligands have been found only in Deuterostomes (Cluster C3 identifies a possible ortholog in *C. teleta* as well), whereas the TGF-β *sensu stricto* and myostatin, activin and inhibin ligands arose from more ancient duplications [62, 63]. Furthermore, the representatives from the myostatin cluster are found in more protostome and non-bilaterian lineages than the TGF-β *sensu stricto* ligands. Thus, each cluster represents a set of TGF-β-like ligands that exhibit both sequence similarity and distinct evolutionary origins.

The other class of TGF-β ligands is the BMP-like ligands, represented by the human disease genes in Cluster C4. In each of the non-Deuterostome species studied here, Cluster C4 contains at most one ortholog to the full set of human BMP-like ligand disease genes. Generally, that ortholog is an RBH to *BMP2*, but not always. This is a case where duplications in the earliest Deuterostomes are not determined by the clustering to create a new, unique subfamily of paralogous genes, and using RBH relationships alone would not reveal this level of detail regarding gene family evolution.

The TGF-β receptor genes are generally classified as type I or type II; our human disease gene set includes only the type II receptors in Cluster C5. This cluster also contains the human non-disease gene *ACVR2A* (data not shown). We note that a TGF-β type II receptor was previously identified in *M. leidyi* [62], but was an outgroup to the rest of the gene family, explaining why it was likely excluded from this cluster.

Lastly, the SMAD proteins are important to the TGF-β signaling pathway. Our human disease gene set contains five *SMAD* genes that are grouped into three meaningful clusters. The inhibitory I-SMADs (*SMAD6* and *SMAD7*) form Clusters C6 and C7, whereas the receptor-associated SMADs (R-SMADs) and common-mediator SMADs (Co-SMADs) group in Cluster C8. This clustering is logical because the non-inhibitory R-SMADs and Co-SMADs have multiple orthologs in the earliest metazoans [62], suggesting the entire class is defined by its early origins opposed to more recent duplications and/or divergence. In addition to the human disease genes shown, Cluster C8 also contains other non-disease-associated SMADs: *SMAD2*, *SMAD5*, and *SMAD9* (data not shown). On the other hand, the inhibitory I-SMADs in Clusters C6 and C7 are similar to each other (and distinct from the R/Co-SMADs), but have different evolutionary origins. *SMAD6* has distant orthologs in many protostomes and non-bilaterian metazoans [62], but *SMAD7* appears to be a paralog of *SMAD6* that arose from a duplication in the early vertebrates. In this case, the clusters separate the two into distinct, meaningful classes of I-SMADs.

Finally, we highlight one potential drawback of our clusters based on Cluster C8. An ortholog is identified in both of the non-metazoans *C. owczarzaki* and *M. brevicollis*, although these species are not known to have any SMAD proteins [62]. Upon further inspection, this cluster includes the *A. queenslandica* predicted protein Aqu1.225807, which appears to be a SMAD protein merged with a Translin protein. This likely represents an incorrectly predicted protein model fusing two separate genes, or alternatively, a novel protein not seen in any other species. As a result, the Translin family of proteins was grouped with the SMAD proteins in this cluster, resulting in orthologs being identified in these non-metazoans to the human Translin gene (data not shown). Thus, using a combined sequence similarity and phylogenetic tree-based clustering method is inherently sensitive to cases where a single inaccurately predicted protein can affect the entire family classification. Fortunately, these situations are uncommon, but nonetheless demonstrate the importance of using high-quality sequence data from well-annotated genome projects. It also highlights the utility of considering the RBH results as complementary to our clusters, because the non-metazoan Translin orthologs are not RBHs to the SMAD proteins in the human disease gene set.
